# Atmospheric Boundary Layer Control on Forest Thermal Properties

**DOI:** 10.1111/gcb.70841

**Published:** 2026-04-07

**Authors:** Matteo Detto, Christopher Still, Amilcare Porporato

**Affiliations:** ^1^ High Meadows Environmental Institute Princeton University Princeton New Jersey USA; ^2^ Smithsonian Tropical Research Institute Balboa Republic of Panama; ^3^ Department of Forest Ecosystems and Society Oregon State University Corvallis Oregon USA; ^4^ Department of Civil and Environmental Engineering Princeton University Princeton New Jersey USA

**Keywords:** atmospheric boundary layer, canopy temperature, land‐atmosphere interactions, thermoregulation, water use

## Abstract

Forest canopy, air temperatures and air humidity ($T_{\mathrm{can}}$, $T_{\mathrm{air}}$, and $q_{\mathrm{air}}$) play a central rol in regulating energy and gas exchange between vegetation and the atmosphere. Although often treated as independent drivers of canopy processes, $T_{\mathrm{air}}$ and $q_{\mathrm{air}}$ are dynamically coupled to $T_{\mathrm{can}}$ via surface energy fluxes and atmospheric boundary layer (ABL) development. We investigated how plant physiology mediates this coupling. Using data from a tropical ecosystem, we studied a process‐based forest model dynamically coupled with an ABL growth model to simulate diurnal interactions between the canopy and the atmosphere. We systematically varied plant traits related to water use and thermal regulation to assess their effects on $T_{\mathrm{can}}\sim T_{\mathrm{air}}$ coupling and feedback. We focused on three metrics: the slope of the $T_{\mathrm{can}}\sim T_{\mathrm{air}}$ relationship, the peak of $T_{\mathrm{can}}$ reached during the day and the lag between the maximum $T_{\mathrm{air}}$ and $T_{\mathrm{can}}$, indicating hysteresis. Conservative water use, by reducing transpiration, leads to greater canopy warming, which intensifies sensible heat flux and accelerates ABL growth. This, in turn, raises near‐surface air temperature and vapor pressure deficit (VPD), amplifying thermal and water stress. In contrast, greater water use enhances evaporative cooling and slows ABL development, thereby moderating these feedback. Surprisingly, the slope of the $T_{\mathrm{can}}\sim T_{\mathrm{air}}$ relationship is quite insensitive to plant water‐use syndromes. This insight extends beyond modeling. Empirical studies often treat $T_{\mathrm{air}}$ and VPD as independent drivers of transpiration, photosynthesis, or stomatal conductance. Our results challenge this assumption, showing that these variables are influenced by plant function itself. $T_{\mathrm{can}}$ is not a passive outcome but an active mediator of energy, water, and carbon exchange, regulated by a feedback loop involving leaf physiology and atmospheric dynamics. Studies using $T_{\mathrm{can}}$ or the $T_{\mathrm{can}}\sim T_{\mathrm{air}}$ relationship—whether from remote sensing or field data—as a proxy for forest stress or function, must account for this coupling.

## Graphical abstract

Forests do not just respond to weather, they actively shape it. Using a model of tropical forests, we show that how plants use water influences how much they heat the air above them: water‐saving plants warm the canopy and atmosphere, while water‐use strategies that promote evaporation help keep both cooler. This challenges the common assumption that air temperature and humidity act independently, revealing instead a feedback loop between plant function and the atmosphere that affects how forests respond to climate stress.

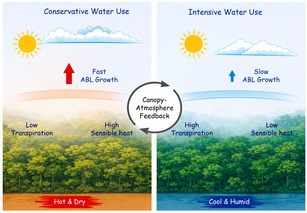

## 1 Introduction

Forests play a fundamental role in the Earth system, driving global water and carbon cycles and contributing to climate regulation and mitigation (Bonan 2008). At the interface between vegetation and atmosphere, canopy temperature emerges as a key state variable that mediates numerous biophysical processes—from leaf metabolism and photosynthetic efficiency to stomatal regulation and surface energy exchange (Alkama and Cescatti 2016; Monteith and Unsworth 2013). Canopy temperature integrates multiple environmental drivers and plant responses, making it a powerful indicator of forest functioning and stress (Javadian et al. 2024; Still et al. 2021).

In both empirical and modeling studies, canopy temperature is commonly analyzed in relation to air temperature and humidity. For example, canopy temperature observations from remote sensing platforms (thermocameras, satellites, infrared sensors) have prompted studies relating $T_{\mathrm{can}}$ and $T_{\mathrm{air}}$ and interpreting the slope and hysteresis as a measure of biological thermoregulation (e.g., Gimenez et al. 2019; Guo et al. 2023; Miller et al. 2021; Pau et al. 2018; Still et al. 2022). However, these atmospheric variables are often treated as independent drivers. This widespread assumption overlooks a fundamental dynamic link: the feedback between the surface energy fluxes and the development of the atmospheric boundary layer (ABL). Sensible and latent heat released by the canopy drive daytime ABL growth, which in turn modifies near‐surface air temperature and humidity, creating a feedback loop between the forest canopy and the atmosphere (Helbig et al. 2016; Panwar et al. 2019; Stull 1988).

Previous work has recognized the importance of ABL dynamics for land‐atmosphere interactions (Helbig et al. 2021; Margulis and Entekhabi 2001; Rey‐Sanchez et al. 2021); however, the feedback mechanisms linking these dynamics to plant water use and canopy thermal properties have not been explicitly studied. Most modeling frameworks and statistical analyses implicitly assume that air temperature and humidity are exogenous, examining how canopy temperature responds without considering that it also interacts with the surrounding atmosphere. Since canopy sensible and latent heat fluxes depend on canopy temperature and in turn alter air temperature and humidity via ABL dynamics, the two temperatures are dynamically coupled, not independent.

To illustrate the issue, consider an analogous case in ecohydrology (Porporato and Yin 2022). Soil moisture and transpiration are tightly coupled: water loss through transpiration depletes soil water available to roots, which in turn constrains further loss via declining water status and stomatal closure. Prescribing soil moisture as an external constraint ignores this feedback and can lead to incomplete or even misleading conclusions, except in diagnostic contexts—typically models constrained by observations—for example, to infer which plant traits or model parameters best reproduce observed ecosystem water and carbon fluxes.

A similar logic applies to canopy and air temperature. Their interaction is mediated by turbulent energy exchange and ABL dynamics. Overlooking this coupling may obscure how different plant traits influence water loss and heat dissipation and thus affect the observed $T_{\mathrm{can}}\sim T_{\mathrm{air}}$ relationship and ultimately influence forest water and carbon cycling.

This feedback operates across multiple levels. First, canopy temperature directly affects photosynthetic and respiratory processes due to their temperature sensitivity. Second, air temperature and humidity jointly determine vapor pressure deficit (VPD), which governs atmospheric water demand and is a key driver of stomatal regulation via its impacts on soil–plant water status. This issue, which also applies to Earth System Models when they are run at the single grid scale (i.e., decoupled from the atmosphere), is not limited to modeling. Many empirical studies treat air temperature or VPD as independent variables in statistical inference, ignoring their feedback with canopy processes.

In this study, we ask: *How do different plant water‐use and heat fluxes modulate the relationship between canopy and air temperatures, given their dynamic coupling through ABL development?*

Answering this question, we show how these two variables are linked via the ABL dynamics, giving rise to a hysteretic branch during their diurnal evolution.

## 2 Methods

We coupled a multilayer soil–plant–atmosphere model with a mixed‐layer ABL model to represent canopy–atmosphere interactions. The forest model (FORCE‐1.0) simulates radiative transfer, leaf energy balance, photosynthesis, and stomatal–hydraulic regulation, while the ABL model resolves boundary‐layer growth, temperature, and humidity. This framework captures the coupled exchange of energy, water vapor, and CO_2_ between forest canopies and the atmosphere under hydro‐climatic stress.

### 2.1 Forest Model

We used a multilayer process‐based model that simulates the exchange of energy, momentum, water vapor, and CO_2_ between each forest canopy layer and the atmosphere, the Forest Canopy Exchange model (FORCE‐1.0; Detto and Pacala 2022).

The model is comprised of four modules: (*i*) radiative transfer throughout the canopy layers, (*ii*) leaf energy budget, (*iii*) leaf photosynthesis, and (*iv*) stomatal optimization sub‐models operating at each canopy layer.

A basic description of FORCE‐1.0 is presented below (for a complete analytical description of the model and its parametrization, please refer to the Supporting Information S1).

- The radiative transfer model used a two‐stream approximation to represent the penetration, absorption, and scattering of direct and diffuse radiation (both downward and upward) through the canopy profile in the visible (VIS), near‐infrared (NIR), and thermal bands (Meador and Weaver 1980; Pinty et al. 2006). For each band, the absorption and scattering coefficients were parameterized from leaf optical properties (reflectance, transmittance, and emissivity) and leaf angle distributions (Yang et al. 2023; Yuan et al. 2017). The absorbed radiation, on both sides of the leaf, in the VIS was used to compute the photosynthetic rate (Béland and Baldocchi 2021; De Pury and Farquhar 1997), and the sum of all absorbed short‐ and longwave radiation was used to compute the leaf energy budget. The absorbed radiation was computed separately for shaded and sunlit leaves (Norman 1980). Sunlit leaves were further grouped according to their relative orientation to the sun's direction throughout the course of the day. The frequency of leaf orientation was computed from leaf angle distributions, assuming a more erectophile distribution at the canopy top and a planophile at the bottom.
- The leaf temperature $T_{L}$ in each canopy layer i and exposure class j is obtained by a leaf energy budget expressed as:

(1) $$R_{a,\mathrm{ij}}=R_{L,\mathrm{ij}}+\lambda E_{\mathrm{ij}}+H_{\mathrm{ij}}$$

where $R_{a,\mathrm{ij}}$ is the sum of all radiation fluxes absorbed by a leaf on both side in the layer‐*i* in the exposure class j, $R_{L,\mathrm{ij}}$ is the long‐wave radiation emitted by the leaf, $E_{\mathrm{ij}}$ and $H_{\mathrm{ij}}$ are the evaporation and sensible heat per unit of one‐sided leaf area. Latent and sensible heat were parametrized with leaf boundary layer resistances and stomatal conductance following (Campbell and Norman 1998) assuming an exponential wind profile within the canopy (Yi 2008). The emitted radiation was computed with the Stefan‐Boltzmann law. All terms in the RHS of (1) are nonlinear functions of $T_{L}$.

iii Net photosynthesis per unit of leaf area, $A_{n}$, was modelled via Michaelis–Menten type dependence upon CO_2_ concentration in the mesophyll (Farquhar et al. 1980). The kinetic variables were parameterized using temperature response functions (Bernacchi et al. 2003; Medlyn et al. 2002) and empirical relationships among maximum carboxylation velocity ($V_{c,\max}$), maximum rate of electron transport ($J_{\max}$) and leaf dark respiration ($R_{d}$) (Atkin et al. 2015; Walker et al. 2014). Intercellular CO_2_ concentration was computed from ambient CO_2_ (fixed at 400 ppm) via Fick's law.
iv A stomatal optimization scheme coupled with a hydraulic model was implemented to represent stomatal response to light, vapor pressure deficit, leaf temperature and soil water potential on the condition of carbon gain maximization, formulated as follows:

(2) $$\max_{g_{s}}\left(A_{n}-\Theta \right)$$

where $g_{s}$ is the stomatal conductance and Θ is the cost of water consumption, such as embolism and dehydration (Cowan and Farquhar 1977; Katul et al. 2010). We assume Θ is a nonlinear function of leaf water potential $\psi_{L}$ and proportional to one minus the loss of leaf hydraulic conductivity (Bartlett et al. 2019; Wolf et al. 2016):

(3) $$\Theta =c_{w}\left(1-\frac{K_{p}\left(\psi_{L}\right)}{K_{\max}}\right)$$

where $K_{p}$ is the whole‐plant hydraulic conductance per unit of leaf area and $c_{w}$ is an empirical parameter. $\psi_{L}$ is determined by integrating Darcy's law along the whole plant hydraulic path, as follows:

(4) $$E=-\int_{{\bar{\psi }}_{s}}^{\psi_{L}}K_{p}\left(\psi \right)\mathrm{d\psi}$$

where ${\bar{\psi }}_{s}$ is the root‐weighted average soil water potential.

### 2.2 ABL Growth and Surface Coupling

The height of the ABL (h), the mean potential temperature (θ) and specific humidity (q) are expressed by the following system of differential equations (Stull 1988; Vilà‐Guerau de Arellano et al. 2015):

(5) $$\left\{\begin{array}{c} \frac{\mathrm{dh}}{\mathrm{dt}}=w_{e}+w_{s}\;\\ \frac{\mathrm{d\theta}}{\mathrm{dt}}=\frac{{\bar{w^{\prime }\theta^{\prime }}}_{s}-{\bar{w^{\prime }\theta^{\prime }}}_{e}}{h}\\ \frac{\mathrm{dq}}{\mathrm{dt}}=\frac{{\bar{w^{\prime }q^{\prime }}}_{s}-{\bar{w^{\prime }q^{\prime }}}_{e}}{h} \end{array}\right.$$

where $w_{e}$ and $w_{s}$ is the entrainment and subsidence velocities, ${\bar{w^{\prime }\theta^{\prime }}}_{s}$ and ${\bar{w^{\prime }q^{\prime }}}_{s}$ are the surface temperature and water flux, ${\bar{w^{\prime }\theta^{\prime }}}_{e}$ and ${\bar{w^{\prime }q^{\prime }}}_{e}$ are the entrainment fluxes (see Appendix A for parametrization of these variables).

The link between θ and q and the air and humidity at the canopy top $T_{\mathrm{air}}$ and $q_{\mathrm{air}}$ is given by bulk transfer relationships in the surface layer

(6a) $${\bar{w^{\prime }\theta^{\prime }}}_{s}=\frac{T_{\mathrm{air}}-\theta }{r_{\mathrm{aH}}}$$

(6b) $${\bar{w^{\prime }q^{\prime }}}_{s}=\frac{q_{\mathrm{air}}-q}{r_{\mathrm{aq}}}$$

where the aerodynamics resistances $r_{\mathrm{aH}}$ and $r_{\mathrm{aq}}$ are parameterized using Monin‐Obukhov similarity theory (Appendix A). Equations ((5), (6a), (6b)) are coupled to the surface fluxes and solved by the FORCE model:

(7) $$\left[{\bar{w^{\prime }\theta^{\prime }}}_{s},{\bar{w^{\prime }q^{\prime }}}_{s},T_{\mathrm{can}}\right]=\text{FORCE}\left(T_{\mathrm{air}},q_{\mathrm{air}}\right|R_{\mathrm{sw}},R_{\mathrm{lw}},f_{D},z_{e},U,{\bar{\psi }}_{s})$$

Together, Equations ((5), (6a), (6b), (7)) constitute a system of differential‐algebraic nonlinear equations that are solved numerically. To speed up numerical computation, FORCE is replaced by an emulator that has been previously trained using an Artificial Neural Network (ANN). The emulator shows excellent agreement with the solution of the model, with R^2^ greater than 0.99 (Figure S3).

**FIGURE 1:**
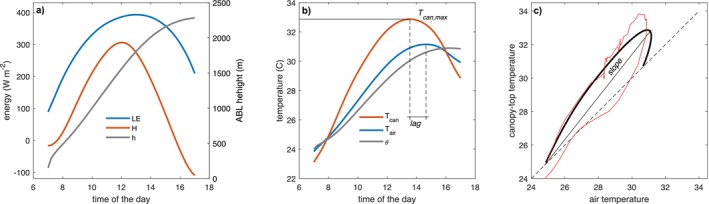
Examples of simulated atmospheric boundary layer dynamics coupled with a canopy forest model. (a) Latent heat (LE) and sensible heat (H) and ABL height (h) above the soil surface as a function of time of day. (b) Air temperature at canopy top and canopy‐top temperature (average leaf temperature of the first leaf layer) as function of time of day. (c) Relationship between canopy‐top temperature and air temperature. As an example, a real pattern observed at Barro Colorado Island on April 16, 2017 is shown (red line).

Canopy‐top temperature (simply $T_{\mathrm{can}}$) was defined as the average leaf temperature in the uppermost canopy layer corresponding to a cumulative leaf area index of one. It is intended to represent the forest temperature observed by remote sensing platforms such as satellites, thermal cameras, or infrared thermometers. $T_{\mathrm{air}}$ and θ denote the average air temperature within the canopy space and across the entire ABL, respectively. An example of the diurnal dynamics of these three temperatures is shown in Figure 1b.

### 2.3 Observational Data

We parameterized the model with data from a semi‐deciduous tropical forest on Barro Colorado Island (BCI, 9°9′25.44″ N, 79°50′54.65″ W) in the Panama Canal Zone, where long‐term eddy covariance measurements are available (Detto and Pacala 2022). The site has a tropical monsoon climate (Köppen–Geiger classification) with mean annual rainfall of 2640 mm, a pronounced dry season from mid‐December to mid‐April, and a mean annual temperature of 26°C with little seasonal variation. The forest has a mean canopy height of ~30 m, a leaf area index of ~6, and an aboveground biomass of ~150 Mg C ha^−1^ (Muller‐Landau et al. 2014).

We first tuned FORCE by parameterizing vegetation traits using available observations, including leaf angle distribution and leaf optical properties (Serbin et al. 2021), and calibrating key parameters—namely, maximum carboxylation velocity and stomatal sensitivity to leaf water potential—against observed sensible heat, latent heat, gross primary production (GPP) from the eddy covariance data, and canopy‐top temperatures measured by infrared thermometers (SI‐400, Apogee Instruments, Logan, UT, USA). The model was able to reasonably reproduce the observed fluxes and canopy‐top temperature (Figure S4).

The diurnal variation of external drivers for the FORCE model—including incoming solar radiation, incoming longwave radiation, wind speed, friction velocity, solar zenith angle, and the fraction of diffuse radiation—was derived by averaging all available data from 2012 to 2017 during the dry season (mid‐December to mid‐April). Only days with low cloud cover were included, defined as those when daily diffuse radiation was less than 50% of total incoming radiation (Figure S5). This selection was made to focus on periods when the forest experiences maximum hydro‐thermal stress, which is particularly relevant for assessing risks to forest health, and when the $T_{\mathrm{can}}-$ ABL coupling is most expected.

ABL temperature and humidity profiles were obtained from atmospheric soundings at the nearby Albrook Airport. Lapse rates were estimated by performing a linear fit on the available data up to 5 km altitude during the dry season, as defined above (Figure S1).

### 2.4 Simulations

We performed a series of simulations to investigate the drivers of canopy‐air temperature ($T_{\mathrm{can}}\sim T_{\mathrm{air}}$) coupling and its biological and environmental controls.

#### 2.4.1 Simulations Without Biological Control of ABL Dynamics

In this set of simulations, we used a model with constant stomatal conductance ($g_{s}$) throughout the day. A fixed value of realistic $g_{s}$ was assigned at the canopy top and decreased linearly along the canopy profile. We conducted 30 independent simulations, varying $g_{s}$ from a canopy‐top value of 0.085 to 0.380 mol m^−2^ s^−1^.

Most parameters in the Farquhar model—such as $V_{c,\max}$, $J_{\max}$ and the Michaelis–Menten constants—are temperature dependent (Bernacchi et al. 2003). Changes in leaf temperature, driven by shifts in evaporative cooling, create a feedback loop that alters photosynthetic rates beyond the direct effects of stomatal aperture. To isolate this biological control, we simulated a case with stomatal optimization for carbon gain (Equation 2) but with photosynthetic kinetics fixed at 25°C. Comparing these simulations with those using temperature‐dependent kinetics allows us to assess the role of stomatal regulation in canopy thermoregulation.

#### 2.4.2 Simulations Exploring Stomatal Dynamics

In this set, we varied the water cost parameter of stomatal optimization $c_{w}$ (in Equation 3) from 1 to 4 μmol m^−2^ s^−1^. Higher values of $c_{w}$ impose a greater cost of water loss, prompting plants to adopt more conservative use of water by readily restricting stomatal conductance.

#### 2.4.3 Simulations Without ABL Coupling

In this set, we repeated the water‐saving simulations from Equation (2), but removed ABL coupling by prescribing $T_{\mathrm{air}}$ and $q_{\mathrm{air}}$ as external forcings—a common assumption in ecosystem modeling. We set the diurnal variation of these variables equal to that of the least conservative strategy to examine how a more conservative use of water would behave in the absence of dynamic ABL feedback on these variables.

Finally, we performed a sensitivity analysis on the physical parameters driving ABL growth to identify which environmental factors play a more important role in regulating the canopy–air temperature coupling (maximum $T_{\mathrm{can}}$, lag and slope), including potential temperature and specific humidity lapse rate ($\gamma_{T}$ and $\gamma_{q}$), initial air temperature at the surface before ABL development ($\theta_{0}$), initial ABL height ($h_{0}$), and entrainment ratio β (i.e., the ratio between the top and surface buoyancy fluxes). This analysis was conducted for two cases representing different levels of stomatal responses to water stress. The sensitivity was quantified as:

(8) $$\sigma =\log\frac{y_{1}}{y_{2}}/\log\frac{x_{1}}{x_{2}}$$

where $y_{1}$ and $y_{2}$ are the coupling metrics computed by varying the parameter x from $x_{1}$ to $x_{2}$ and leaving the other parameters unchanged. For each parameter, values were varied by ±5% from their default value.

## 3 Results

Figure 1 illustrates the diurnal dynamics simulated by the coupled forest canopy and ABL growth models. The simulation captures (a) the progressive increase in ABL height driven by surface energy fluxes, and (b) the diurnal variation of three temperatures: air temperature within the canopy, canopy‐top temperature (average leaf temperature of the uppermost leaf layer), and the mean ABL temperature. All three rise rapidly during morning ABL development and decline in the afternoon as surface fluxes weaken, except ABL temperature, which instead plateaus.

The relationship between canopy and air temperatures is further illustrated in Figure 1c, which shows a characteristic hysteresis pattern. This figure also highlights the three key metrics used in this study to quantify the coupling between canopy and air temperatures: (1) the daily maximum canopy temperature, (2) the time lag between the canopy and air temperature maxima, and (3) the slope of the canopy‐air temperature relationship.

**FIGURE 2:**
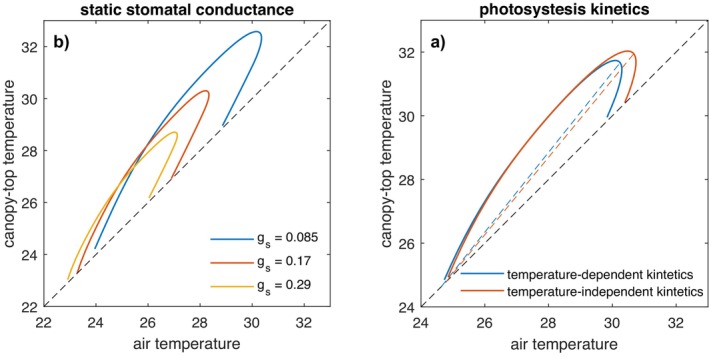
(a) Hysteresis between $T_{\mathrm{can}}$ and $T_{\mathrm{air}}$ for simulations with constant stomatal conductance (mol m^−2^ s^−1^) and (b) for simulations with and without temperature‐dependent kinetics of the photosynthetic apparatus (see Section 2).

Figure 2a shows the relationship between canopy‐top temperature and air temperature from simulations with varying levels of constant stomatal conductance ($g_{s}$). Simulations with more restricted conductance exhibit reduced evapotranspiration and enhanced sensible heat flux, which lead to higher ABL height, and elevated air and canopy‐top temperatures (Figure 2a). However, despite a fivefold variation in $g_{s}$ the slope of the $T_{\mathrm{can}}\sim T_{\mathrm{air}}$ relationship remains relatively insensitive. In contrast, the lag between canopy and air temperature maxima increases with more restricted conductance, reflecting the greater thermal inertia of a deeper ABL.

**FIGURE 3:**
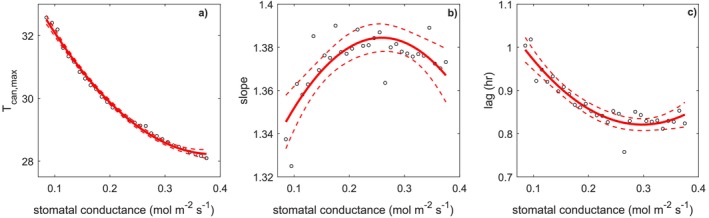
Dependence of maximum canopy temperature (panel a), slope (panel b), and $T_{\mathrm{can}}$ versus Tair peak lag (panel c) on stomatal conductance for simulations with constant diurnal stomatal conductance (i.e., no biological controls on ABL dynamics). Black circles are the simulations, red line are the quadratic regression and confidence intervals (dashed lines).

In Figure 3 these patterns are explored quantitatively. In contrast to the strong nonlinear reduction in maximum $T_{\mathrm{can}}$, the slope is relatively insensitive to a variation in $g_{s}$ (Figure 3b). The lag between canopy and air maxima increases because of ABL inertia as it gets higher (Figure 3c), though also in this case, the trend is relatively weak.

Figure 2b compares simulations with and without temperature‐dependent kinetics of the photosynthetic apparatus. Incorporating temperature dependence leads to a lower maximum $T_{\mathrm{can}}$ (by about 1°C) as the plant enhances transpiration to control optimal temperature for photosynthesis. This simple case shows how incorporating a mechanism of thermoregulation prompts the plant to adopt less conservative water use. Interestingly, the $T_{\mathrm{can}}\sim T_{\mathrm{air}}$ slope is slightly steeper with thermoregulation, which is counterintuitive since one might expect weaker coupling when plants actively regulate temperature. However, the difference is minimal and does not substantially alter the overall relationship.

**FIGURE 4:**
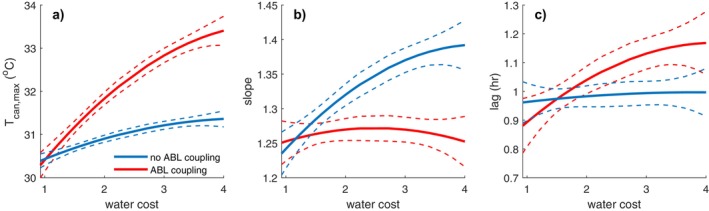
Dependence of maximum canopy temperature (a), slope (b) and lag (c) to water cost for simulations with (red) and without (blue) ABL couplings. Solid lines are the quadratic regression and confidence intervals (dashed lines).

Next, we explored the role of plant water conservation. As in the constant stomatal conductance cases, more conservative water‐use behavior (i.e., higher water costs and thus lower stomatal conductance and transpiration) led to a deeper ABL and higher canopy and air temperatures. When we compared these results to simulations without ABL coupling—where air temperature is prescribed as an independent forcing (set equal to the value from the less water conservative simulation), clear differences emerged. Neglecting ABL coupling substantially altered the patterns of $T_{\mathrm{air}}\sim T_{\mathrm{can}}$ coupling metrics, including the slope, lag, and maximum canopy temperature. Specifically, the simulations with ABL coupling showed a stronger increase in maximum $T_{\mathrm{can}}$ with increasing water conservation, no clear relationship between water cost and slope, and a more pronounced increase in the lag between canopy and air temperature peaks (Figure 4).

**FIGURE 5:**
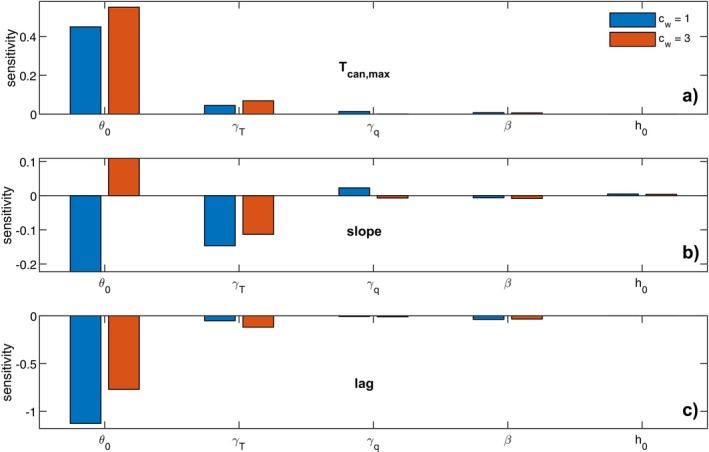
Sensitivity of maximum temperature (a), $T_{\mathrm{can}}/T_{\mathrm{air}}$ slope (b), and $T_{\mathrm{can}}$ peak – $T_{\mathrm{air}}$ peak lag (c) to ABL model parameters for two cases. $\theta_{0}$ is the initial ABL temperature at the surface, $\gamma_{T}$ and $\gamma_{q}$ are the temperature and absolute humidity lapse rate, $h_{0}$ is the initial ABL height and β the entrainment factor (the ratio between the top and surface buoyancy fluxes).

In the final analysis, we study the sensitivity of the three ABL coupling metrics to ABL model parameters (Figure 5). The initial temperature of the ABL is by far the most important parameter, and the temperature lapse rate is the second. However, their relative importance varies for different cases and metrics. Interestingly, for the slope, it switched from negative to positive for the higher water cost scenarios.

**FIGURE 6:**
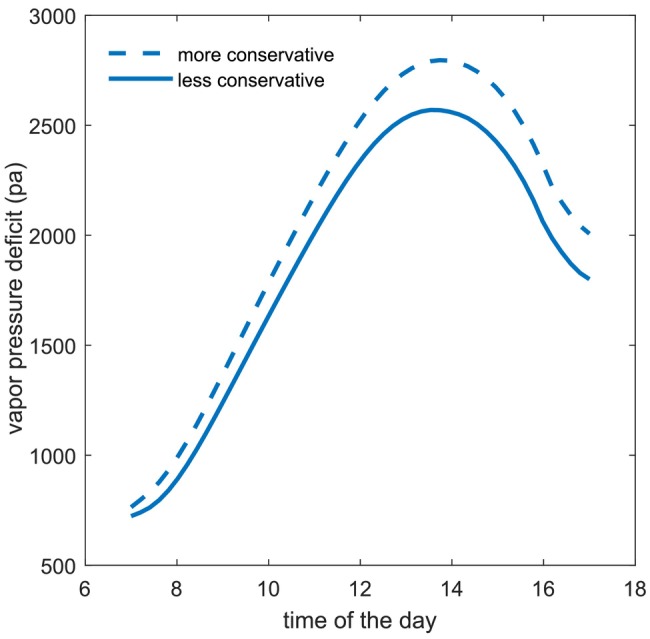
Diurnal variation of vapor pressure deficit determined by the ABL‐forest coupling for two water conserving syndromes.

Although most of the analyses focused on temperature, humidity also plays a critical role in this system. For example, like temperature, vapor pressure deficit also increases in response to higher water cost scenarios. This highlights important negative feedback in forest water regulation: by conserving water, however, plants indirectly also increase atmospheric water demand (Figure 6).

## 4 Discussion

Our study highlights the critical importance of considering the dynamic coupling between forest canopies and the ABL when evaluating canopy thermal properties and forest canopy response to temperature and humidity. We show that plant‐driven fluxes of heat and moisture do not merely respond to atmospheric conditions but actively shape near‐surface air temperature and humidity via ABL development. This coupling feeds back to influence canopy temperatures and, ultimately, forest functions. Therefore, air temperature and humidity are not strictly independent variables but are emergent properties of surface‐atmosphere interactions.

### 4.1 Dynamic Feedback Alters the Canopy‐Air Temperature Relationship

Simulations with varying stomatal conductance revealed a surprising insensitivity of the slope of the $T_{\mathrm{can}}\sim T_{\mathrm{air}}$ relationship, despite a large variation in stomatal conductance. In contrast, maximum canopy temperature and the lag between canopy and air temperature peaks were quite sensitive to plant water use. Conservative water‐use strategies (higher water cost and thus lower $g_{s}$) reduced latent cooling, increased sensible heat flux, and led to a deeper, warmer, and drier boundary layer. These changes resulted in higher maximum canopy temperatures and a longer lag between $T_{\mathrm{can}}$ and $T_{\mathrm{air}}$. These findings highlight that the slope of the $T_{\mathrm{can}}\sim T_{\mathrm{air}}$ relationship might not be the best diagnostic variable for understanding canopy thermal properties.

Interestingly, incorporating temperature‐dependent photosynthetic kinetics prompted plants to modulate transpiration to mitigate thermal stress—effectively engaging in thermoregulation.

### 4.2 Environmental Variability

At our tropical site, temperature variability among days and seasons is comparatively modest. However, in many forested systems subject to stronger synoptic forcing, day‐to‐day variability in ABL dynamics is likely to be substantial and may strongly influence canopy thermal regimes.

As partial insight into how day‐to‐day changes may propagate through the system, the sensitivity analysis of ABL initialization parameters (Figure 6) shows that small differences in early‐morning temperature lead to pronounced variation in canopy–ABL coupling strength. This behavior is analogous to realistic day‐to‐day differences in morning atmospheric profiles and highlights the sensitivity of canopy thermal responses to initial atmospheric conditions. Notably, the effect of baseline air temperature on the slope between $T_{\mathrm{air}}$ and $T_{\mathrm{can}}$ depends on plant water‐use strategy, with conservative and non‐conservative strategies exhibiting distinct responses. These nonlinear interactions further complicate attempts to infer canopy thermal regimes from air temperature alone, underscoring the importance of explicitly accounting for surface–atmosphere coupling.

Cloudiness represents an additional and often dominant source of short‐term environmental variability, as it directly modulates incoming radiation (see Section 4.4). In general, warmer days—typically associated with clear skies and dry conditions—are expected to exhibit stronger canopy–ABL coupling and are therefore most critical for canopy heat stress. Atmospheric subsidence may further amplify these conditions by enhancing surface heating and suppressing vertical mixing, an effect that warrants further investigation (Rey‐Sanchez et al. 2021).

Seasonal changes in moisture availability exert a strong control on canopy–ABL coupling at this site. During the wet season, high soil moisture supports efficient evaporative cooling, limiting sensible heat flux and resulting in weak coupling between canopy temperature and ABL development. In contrast, during the dry season, reduced moisture availability suppresses latent cooling and shifts a larger fraction of available energy into sensible heat, producing deeper ABLs and stronger canopy–atmosphere feedbacks (Figure S2). Thermal stress therefore emerges predominantly under dry‐season conditions, when the canopy becomes more sensitive to atmospheric boundary layer dynamics. This seasonal shift in coupling aligns with the broader paradigm of light limitation versus water limitation that governs tropical forest productivity and phenology (Wagner et al. 2016). Our results add an atmospheric dimension to this framework by showing that transitions between light‐ and water‐limited states—across seasons or environmental gradients—are accompanied by systematic changes in canopy–ABL feedback strength.

### 4.3 Implications for Modeling and Empirical Studies

Our results have several implications for land surface modeling, particularly in Earth System Models (ESMs) run at a single grid, where canopy processes are often decoupled from ABL dynamics. We show that these simplifications can misrepresent plant thermal regimes and evapotranspiration feedback, especially under climate stress. Accurate representation of plant water‐use dynamics, canopy thermal properties, and ABL development is essential to predict how forests will respond to warming, drying, and increasing climate variability.

Neglecting ABL feedback—by prescribing air temperature and humidity as external forcings—altered all key metrics of canopy‐air temperature coupling. In particular, the maximum canopy temperature, the lag, and even the functional form of the $T_{\mathrm{can}}\sim T_{\mathrm{air}}$ relationship differed from the coupled simulations.

A number of recent studies have investigated whether canopy or leaf temperatures track air temperature, and whether forests exhibit consistent thermoregulatory behavior. Guo et al. (2023) conducted a multi‐scale assessment across extratropical ecosystems and found only weak, context‐dependent evidence for ecosystem‐level thermoregulation, concluding that $T_{\mathrm{can}}$ generally follow $T_{\mathrm{air}}$. Still et al. (2022) similarly reported no evidence for canopy‐scale cooling below ambient air temperature across diverse forests, adopting the widely used assumption that $T_{\mathrm{can}}\leq T_{\mathrm{air}}$ and a slope lower than one provides an appropriate reference for detecting thermoregulation. Studies focusing on tropical forests have interpreted temperature responses through the same lens. Miller et al. (2021) showed that only sunlit upper‐canopy leaves exceed $T_{\mathrm{air}}$ and photosynthetic thermal optima, while Pau et al. (2018) evaluated productivity limits using air temperature as the operative thermal environment. Complementary work along climatic gradients reinforces this framework: Zhou et al. (2023) analyzed leaf–air temperature differences to characterize thermal regulation strategies across vegetation types, and Javadian et al. (2024) demonstrated that $T_{\mathrm{can}}$ closely aligns with ecosystem water availability, again interpreting $T_{\mathrm{can}}\sim T_{\mathrm{air}}$ differences relative to a locally measured $T_{\mathrm{air}}$ baseline.

These studies collectively highlight that empirical assessments of canopy thermal behavior often rely on the simplifying assumption that $T_{\mathrm{air}}$ represents an externally imposed boundary condition, against which $T_{\mathrm{can}}$ can be evaluated. However, none of these analyses explicitly consider that the canopy itself can substantially modify the adjacent air mass through radiative and turbulent processes, nor that warming and drying of the air layer immediately above the canopy affects the very benchmark $T_{\mathrm{air}}$ used to diagnose thermoregulation. Our results show that strong canopy–ABL coupling during dry conditions alters both $T_{\mathrm{air}}$ and VPD in ways that feed back onto canopy energy balance. This mechanism reframes how $T_{\mathrm{can}}\sim T_{\mathrm{air}}$ relationships should be interpreted and suggests that deviations from $T_{\mathrm{air}}$ cannot be understood independently of the evolving thermal state of the ABL. Consequently, previous conclusions regarding the presence or absence of thermoregulation should be revisited with explicit attention to canopy–atmosphere coupling, particularly in water‐limited tropical forests where these feedbacks are strongest.

More broadly, the findings suggest that $T_{\mathrm{can}}$ is not merely a diagnostic variable but an active mediator of energy, water, and carbon exchange. Its regulation emerges from a feedback loop involving radiation, leaf physiology, stomatal behavior, and atmospheric dynamics. Future studies aiming to use canopy temperature—whether from remote sensing or field measurements—as a proxy for forest stress or function should account for this coupling.

There is also a key distinction in temporal scales that exacerbates this problem. For example, while soil moisture can often be treated as quasi‐static over diurnal timescales, air temperature and humidity exhibit large fluctuations within a single day. In tropical regions, in particular, the diurnal cycle represents the dominant temperature variation due to minimal seasonality and inter‐daily variability. As a result, neglecting the dynamic coupling between canopy processes and ABL development does not just affect short‐term analyses; it systematically propagates into long‐term studies that use hourly time step data. This oversight can bias interpretations of plant‐climate interactions, even in multi‐year datasets, by implicitly treating air temperature and humidity as external, independent drivers, including studies that assess the impact of VPD on ecosystem productivity.

### 4.4 Cloud Formation and Other Feedback

In this study, we focused on clear‐sky conditions to isolate the effects of canopy–ABL coupling under strong thermal stress. However, as the ABL develops throughout the day, it can reach the lifting condensation level, triggering convective cloud formation. This process is a well‐known land–atmosphere feedback, extensively studied in relation to the hydrological cycle (Cerasoli et al. 2021; Juang et al. 2007; Tuttle and Salvucci 2016; Yin et al. 2015), but its consequences for canopy thermal dynamics remain largely unexplored.

Cloud formation can significantly alter the canopy's radiative environment—reducing total incoming shortwave radiation, increasing incoming longwave radiation, and shifting toward a higher diffuse fraction. These changes can substantially influence leaf energy balance, photosynthesis, and transpiration, and thus represent a critical extension of the canopy–ABL coupling framework toward more realistic and variable sky conditions (Durand et al. 2021; Freedman et al. 2000; Sedlar et al. 2022).

Moreover, scattered cloud passages can further modulate the $T_{\mathrm{can}}\sim T_{\mathrm{air}}$ relationship through ABL inertia. When a cloud casts temporary shade, canopy surfaces cool more rapidly than the overlying air, due to their lower thermal inertia. This can result in short‐term effects where differences between $T_{\mathrm{can}}$ and $T_{\mathrm{air}}$ appears lower than expected or even reversed. Capturing these transient effects is important for interpreting high‐frequency observations and refining canopy energy balance models under partly cloudy conditions.

Other feedback might include CO_2_ depletion, as ABL growth and forest sink will dilute CO_2_ concentrations, with negative feedback on photosynthesis (Tang et al. 2025). In principle, this could be simulated by including CO_2_ dynamics in the ABL model (Equations (5), (6a), (6b)).

### 4.5 Evolutionary Adaptation

The feedback between ABL development and canopy thermal properties raises the question of whether plants have evolved to adapt to ABL dynamics, particularly in stomatal conductance regulation. This hypothesis is plausible: ABL formation has occurred daily throughout the history of terrestrial ecosystems, and stomatal behavior is widely interpreted through optimality principles aimed at maximizing carbon gain under constraints (Cowan and Farquhar 1977; Gardner et al. 2023). Evidence that some tropical tree species exhibit adaptive leaf thermoregulation (Middleby et al. 2025) further suggests that selection can act on traits influencing canopy–atmosphere thermal interactions.

This question becomes especially relevant given the role of VPD, a key indirect driver of stomatal conductance (Meinzer 2002; Oren et al. 1999). Our simulations show that more water‐conservative strategies indirectly also increase VPD, amplifying atmospheric water demand. This finding illustrates an important loop: while plants conserve water by restricting stomata, they may indirectly intensify evaporative demand by warming and drying the ABL—potentially leading to greater long‐term stress. This suggests that ABL coupling may serve as moderating feedback on overly conservative water‐use strategies. Whether plant traits and strategies have evolved, in part, to balance this feedback remains an open question and a promising avenue for future research, considering that rising VPD associated with climate change and other anthropogenic effects (Barkhordarian et al. 2019) is generating growing concern about its impact on plant water use and productivity (Grossiord et al. 2020).

## Author Contributions

**Matteo Detto:** conceptualization, data curation, formal analysis, funding acquisition, investigation, methodology, writing – original draft. **Amilcare Porporato:** supervision, writing – review and editing. **Christopher Still:** writing – review and editing.

## Conflicts of Interest

The authors declare no conflicts of interest.

## Supporting information


**Figure S1:** Potential temperature and specific humidity profiles obtained from morning atmospheric.
**Figure S2:** Mean Diurnal Cycle of PBL height obtained from ERAS5 Reanalysis hourly gridded product (0.25°).
**Figure S3:** Comparison between ecosystem fluxes and 𝑇_*𝑐an*_ obtained from FORCE model simulations and the emulator.
**Figure S4:** Comparison between observed and predicted values of the four variables used to calibrate.
**Figure S5:** Diurnal variation of basic atmospheric forcings used to drive FORCE model. Data are obtained.

**Data S1:** The Forest Canopy Exchange Model—FORCE‐1.0.

**Appendix A.** ABL model parametrization.

## Data Availability

The eddy covariance data are available in the Ameriflux database at https://ameriflux.lbl.gov/sites/siteinfo/PA‐Bar. Wright and Detto (2026), AmeriFlux BASE PA—Bar Barro Colorado Island, Ver. 1‐5, 444 AmeriFlux AMP (Dataset; https://doi.org/10.17190/AMF/3015322). The MATLAB scripts used for the forest‐process model FORCE‐1.0 and the ABL growth model, are available in Zenodo repository: mdetto/FORCE‐1.0: FORCE and ABL growth models (V1.0). Zenodo (https://doi.org/10.5281/zenodo.19117886). All other data, including Canopy temperature, ERA5 PBL height and radiosonde can be dowloaded in the Zenodo repository: Matteo Detto (2026). Atmospheric boundary layer control on forest thermal properties—Dataset (Data set). In Global Change Biology (Zenodo: https://doi.org/10.5281/zenodo.19118385).

## References

[gcb70841-bib-0001] Alkama, R. , and A. Cescatti . 2016. “Climate Change: Biophysical Climate Impacts of Recent Changes in Global Forest Cover.” Science 351, no. 6273: 600–604. 10.1126/science.aac8083. 26912702

[gcb70841-bib-0002] Atkin, O. K. , K. J. Bloomfield , P. B. Reich , et al. 2015. “Global Variability in Leaf Respiration in Relation to Climate, Plant Functional Types and Leaf Traits.” New Phytologist 206, no. 2: 614–636. 10.1111/nph.13253. 25581061

[gcb70841-bib-0003] Barkhordarian, A. , S. S. Saatchi , A. Behrangi , P. C. Loikith , and C. R. Mechoso . 2019. “A Recent Systematic Increase in Vapor Pressure Deficit Over Tropical South America.” Scientific Reports 9, no. 1: 1–12. 10.1038/s41598-019-51857-8. 31653952 PMC6814800

[gcb70841-bib-0004] Bartlett, M. K. , M. Detto , and S. W. Pacala . 2019. “Predicting Shifts in the Functional Composition of Tropical Forests Under Increased Drought and CO2 From Trade‐Offs Among Plant Hydraulic Traits.” Ecology Letters 22, no. 1: 67–77. 10.1111/ele.13168. 30402964

[gcb70841-bib-0005] Béland, M. , and D. D. Baldocchi . 2021. “Vertical Structure Heterogeneity in Broadleaf Forests: Effects on Light Interception and Canopy Photosynthesis.” Agricultural and Forest Meteorology 307: 108525. 10.1016/J.AGRFORMET.2021.108525.

[gcb70841-bib-0006] Bernacchi, C. J. , C. Pimentel , and S. P. Long . 2003. “In Vivo Temperature Response Functions of Parameters Required to Model RuBP‐Limited Photosynthesis.” Plant, Cell and Environment 26, no. 9: 1419–1430. 10.1046/j.0016-8025.2003.01050.x.

[gcb70841-bib-0007] Bonan, G. B. 2008. “Forests and Climate Change: Forcings, Feedbacks, and the Climate Benefits of Forests.” Science (New York, N.Y.) 320, no. 5882: 1444–1449. 10.1126/science.1155121. 18556546

[gcb70841-bib-0008] Campbell, G. , and J. M. Norman . 1998. “An Introduction to Environmental Biophysics.” Journal of Environmental Quality 6, no. 4: 474. 10.2134/jeq1977.00472425000600040036x.

[gcb70841-bib-0009] Cerasoli, S. , J. Yin , and A. Porporato . 2021. “Cloud Cooling Effects of Afforestation and Reforestation at Midlatitudes.” Proceedings of the National Academy of Sciences of the United States of America 118, no. 33: e2026241118. 10.1073/PNAS.2026241118. 34373327 PMC8379994

[gcb70841-bib-0010] Cowan, I. R. , and G. D. Farquhar . 1977. “Stomatal Function in Relation to Leaf Metabolism and Environment.” Symposia of the Society for Experimental Biology 31, no. 1973: 471–505. 756635

[gcb70841-bib-0011] De Pury, D. G. G. , and G. D. Farquhar . 1997. “Simple Scaling of Photosynthesis From Leaves to Canopies Without the Errors of Big‐Leaf Models.” Plant, Cell and Environment 20, no. 5: 537–557. 10.1111/j.1365-3040.1997.00094.x.

[gcb70841-bib-0013] Detto, M. , and S. W. Pacala . 2022. “Plant Hydraulics, Stomatal Control and the Response of a Tropical Forest to Water Stress Over Multiple Temporal Scales.” Global Change Biology 28: 4359–4376. 10.1111/gcb.16179. 35373899

[gcb70841-bib-0059] Detto, M. 2026. Atmospheric boundary layer control on forest thermal properties ‐ Dataset [Data set]. Zenodo. 10.5281/ZENODO.19118384.41947508

[gcb70841-bib-0014] Durand, M. , E. H. Murchie , A. V. Lindfors , O. Urban , P. J. Aphalo , and T. M. Robson . 2021. “Diffuse Solar Radiation and Canopy Photosynthesis in a Changing Environment.” Agricultural and Forest Meteorology 311: 108684. 10.1016/J.AGRFORMET.2021.108684.

[gcb70841-bib-0015] Farquhar, G. D. , S. Von Caemmerer , and J. A. Berry . 1980. “A Biochemical Model of Photosynthetic CO2 Assimilation in Leaves of C3 Species.” Planta 149: 78–90. 10.1007/BF00386231. 24306196

[gcb70841-bib-0016] Freedman, J. M. , D. R. Fitzjarrald , K. E. Moore , and R. K. Sakai . 2000. “Boundary Layer Clouds and Vegetation‐Atmosphere Feedbacks.” Journal of Climate 14: 180–196. www.noaa.ncdc.com.

[gcb70841-bib-0017] Gardner, A. , M. Jiang , D. S. Ellsworth , et al. 2023. “Optimal Stomatal Theory Predicts CO2 Responses of Stomatal Conductance in Both Gymnosperm and Angiosperm Trees.” New Phytologist 237, no. 4: 1229–1241. 10.1111/NPH.18618. 36373000

[gcb70841-bib-0058] Gimenez, B. O. , K. J. Jardine , N. Higuchi , et al. 2019. “Species‐Specific Shifts in Diurnal Sap Velocity Dynamics and Hysteretic Behavior of Ecophysiological Variables During the 2015–2016 El Niño Event in the Amazon Forest.” Frontiers in Plant Science 10. 10.3389/fpls.2019.00830. PMC6611341 31316536

[gcb70841-bib-0019] Grossiord, C. , T. N. Buckley , L. A. Cernusak , et al. 2020. “Plant Responses to Rising Vapor Pressure Deficit.” New Phytologist 226, no. 6: 1550–1566. 10.1111/nph.16485. 32064613

[gcb70841-bib-0020] Guo, Z. , C. J. Still , C. K. F. Lee , et al. 2023. “Does Plant Ecosystem Thermoregulation Occur? An Extratropical Assessment at Different Spatial and Temporal Scales.” New Phytologist 238, no. 3: 1004–1018. 10.1111/nph.18632. 36495263

[gcb70841-bib-0021] Helbig, M. , T. Gerken , E. R. Beamesderfer , et al. 2021. “Integrating Continuous Atmospheric Boundary Layer and Tower‐Based Flux Measurements to Advance Understanding of Land‐Atmosphere Interactions.” Agricultural and Forest Meteorology 307: 108509. 10.1016/J.AGRFORMET.2021.108509.

[gcb70841-bib-0022] Helbig, M. , K. Wischnewski , N. Kljun , et al. 2016. “Regional Atmospheric Cooling and Wetting Effect of Permafrost Thaw‐Induced Boreal Forest Loss.” Global Change Biology 22, no. 12: 4048–4066. 10.1111/gcb.13348. 27153776

[gcb70841-bib-0023] Javadian, M. , R. L. Scott , W. Woodgate , A. D. Richardson , M. P. Dannenberg , and W. K. Smith . 2024. “Canopy Temperature Dynamics Are Closely Aligned With Ecosystem Water Availability Across a Water‐ to Energy‐Limited Gradient.” Agricultural and Forest Meteorology 357: 110206. 10.1016/J.AGRFORMET.2024.110206.

[gcb70841-bib-0024] Juang, J.‐Y. , A. Porporato , P. C. Stoy , et al. 2007. “Hydrologic and Atmospheric Controls on Initiation of Convective Precipitation Events.” Water Resources Research 43, no. 3: W03421. 10.1029/2006WR004954.

[gcb70841-bib-0025] Katul, G. , S. Manzoni , S. Palmroth , and R. Oren . 2010. “A Stomatal Optimization Theory to Describe the Effects of Atmospheric CO2 on Leaf Photosynthesis and Transpiration.” Annals of Botany 105, no. 3: 431–442. 10.1093/aob/mcp292. 19995810 PMC2826246

[gcb70841-bib-0026] Margulis, S. A. , and D. Entekhabi . 2001. “Feedback Between the Land Surface Energy Balance and Atmospheric Boundary Layer Diagnosed Through a Model and Its Adjoint.” Journal of Hydrometeorology 2: 599–620.

[gcb70841-bib-0027] Meador, W. E. , and W. R. Weaver . 1980. “Two‐Stream Approximation to Radiative Transfer in Planetary Atmospheres: A Unified Description of Existing Methods and a New Improvement.” Journal of the Atmospheric Sciences 37, no. 3: 630–643. 10.1175/1520-0469.

[gcb70841-bib-0028] Medlyn, B. E. , E. Dreyer , D. Ellsworth , et al. 2002. “Temperature Response of Parameters of a Biochemically Based Model of Photosynthesis. II. A Review of Experimental Data.” Plant, Cell & Environment 25: 1167–1179.

[gcb70841-bib-0029] Meinzer, F. C. 2002. “Co‐Ordination of Vapour and Liquid Phase Water Transport Properties in Plants.” Plant, Cell and Environment 25, no. 2: 265–274. 10.1046/J.1365-3040.2002.00781.X. 11841669

[gcb70841-bib-0030] Middleby, K. B. , R. Jordan , A. W. Cheesman , et al. 2025. “Local Adaptation Drives Leaf Thermoregulation in Tropical Rainforest Trees.” Global Change Biology 31, no. 9: e70461. 10.1111/GCB.70461. 40905154 PMC12409605

[gcb70841-bib-0031] Miller, B. D. , K. R. Carter , S. C. Reed , T. E. Wood , and M. A. Cavaleri . 2021. “Only Sun‐Lit Leaves of the Uppermost Canopy Exceed Both Air Temperature and Photosynthetic Thermal Optima in a Wet Tropical Forest.” Agricultural and Forest Meteorology 301–302: 108347. 10.1016/j.agrformet.2021.108347.

[gcb70841-bib-0032] Monteith, J. L. , and M. H. Unsworth . 2013. Fundamental Principles of Environmental Physics. Academic Press, Oxford. 10.1007/9783030690250.

[gcb70841-bib-0033] Muller‐Landau, H. C. , M. Detto , R. A. Chisholm , S. P. Hubbell , and R. Condit . 2014. “Detecting and Projecting Changes in Forest Biomass From Plot Data.” In Forests and Global Change, edited by D. A. Coomes , D. F. R. P. Burslem , and W. D. Simonson , 381–416. Cambridge University Press.

[gcb70841-bib-0034] Norman, J. M. 1980. “Interfacing Leaf and Canopy Light Interception Models.” In Predicting Photosynthesis for Ecosystem Models, vol. 2, 1st ed., 49–68. CRC Press. 10.1201/9781351075954.

[gcb70841-bib-0035] Oren, R. , J. S. Sperry , G. G. Katul , et al. 1999. “Survey and Synthesis of Intra‐ and Interspecific Variation in Stomatal Sensitivity to Vapour Pressure Deficit.” Plant, Cell and Environment 22, no. 12: 1515–1526. 10.1046/j.1365-3040.1999.00513.x.

[gcb70841-bib-0036] Panwar, A. , A. Kleidon , and M. Renner . 2019. “Do Surface and Air Temperatures Contain Similar Imprints of Evaporative Conditions?” Geophysical Research Letters 46, no. 7: 3802–3809. 10.1029/2019GL082248.

[gcb70841-bib-0037] Pau, S. , M. Detto , Y. Kim , and C. J. Still . 2018. “Tropical Forest Temperature Thresholds for Gross Primary Productivity.” Ecosphere 9, no. 7: 1–12. 10.1002/ecs2.2311.38357012

[gcb70841-bib-0038] Pinty, B. , T. Lavergne , R. E. Dickinson , J. L. Widlowski , N. Gobron , and M. M. Verstraete . 2006. “Simplifying the Interaction of Land Surfaces With Radiation for Relating Remote Sensing Products to Climate Models.” Journal of Geophysical Research‐Atmospheres 111, no. 2: 1–20. 10.1029/2005JD005952.20411040

[gcb70841-bib-0039] Porporato, A. , and J. Yin . 2022. “Stochastic Soil Moisture Dynamics.” In Ecohydrology: Dynamics of Life and Water in the Critical Zone, 187–224. Cambridge University Press. 10.1017/9781108886321.

[gcb70841-bib-0040] Rey‐Sanchez, C. , S. Wharton , J. Vilà‐Guerau de Arellano , et al. 2021. “Evaluation of Atmospheric Boundary Layer Height From Wind Profiling Radar and Slab Models and Its Responses to Seasonality of Land Cover, Subsidence, and Advection.” Journal of Geophysical Research: Atmospheres 126: e2020JD033775. 10.1029/2020JD033775.

[gcb70841-bib-0041] Sedlar, J. , L. D. Riihimaki , D. D. Turner , et al. 2022. “Investigating the Impacts of Daytime Boundary Layer Clouds on Surface Energy Fluxes and Boundary Layer Structure During CHEESEHEAD19.” Journal of Geophysical Research: Atmospheres 127: e2021JD036060. 10.1029/2021JD036060.

[gcb70841-bib-0042] Serbin, S. , K. Ely , A. Rogers , et al. 2021. “Leaf Spectra, Feb2016‐April2016, PA‐SLZ, PA‐PNM, PA‐BCI: Panama.” 10.15486/NGT/1478523.

[gcb70841-bib-0043] Still, C. J. , G. Page , B. Rastogi , et al. 2022. “No Evidence of Canopy‐Scale Leaf Thermoregulation to Cool Leaves Below Air Temperature Across a Range of Forest Ecosystems.” PNAS 1–8: e2205682119. 10.1073/pnas.2205682119. PMC9499539 36095211

[gcb70841-bib-0044] Still, C. J. , B. Rastogi , G. F. M. Page , et al. 2021. “Imaging Canopy Temperature: Shedding (Thermal) Light on Ecosystem Processes.” In New Phytologist, vol. 230, 1746–1753. John Wiley and Sons Inc. 10.1111/nph.17321. 33666251

[gcb70841-bib-0045] Stull, R. B. 1988. An Introduction to Boundary Layer Meteorology. Springer Netherlands. 10.1007/978-94-009-3027-8/COVER.

[gcb70841-bib-0046] Tang, W. , F. Zhang , P. C. Stoy , R. L. Scott , A. C. I. Tang , and Z. Fu . 2025. “Contribution of Carbon Dioxide Concentration to the Diurnal Variation in Land Surface Carbon Dioxide Uptake From the Atmosphere.” Plant, Cell and Environment 48, no. 9: 6722–6733. 10.1111/pce.15638. 40421681

[gcb70841-bib-0047] Tuttle, S. , and G. Salvucci . 2016. “Atmospheric Science: Empirical Evidence of Contrasting Soil Moisture‐Precipitation Feedbacks Across the United States.” Science 352, no. 6287: 825–828. 10.1126/SCIENCE.AAA7185. 27174987

[gcb70841-bib-0060] Vilà‐Guerau de Arellano, J. , C. C. van Heerwaarden , B. J. H. van Stratum , and K. van den Dries . 2015. “Atmospheric Boundary Layer: Integrating Air Chemistry and Land Interactions.” In Atmospheric Boundary Layer. Cambridge University Press. 10.1017/cbo9781316117422.003.

[gcb70841-bib-0049] Wagner, F. H. , B. Hérault , D. Bonal , et al. 2016. “Climate Seasonality Limits Leaf Carbon Assimilation and Wood Productivity in Tropical Forests.” Biogeosciences 13, no. 8: 2537–2562. 10.5194/bg-13-2537-2016.

[gcb70841-bib-0050] Walker, A. P. , A. P. Beckerman , L. Gu , et al. 2014. “The Relationship of Leaf Photosynthetic Traits ‐ Vcmax and Jmax ‐ to Leaf Nitrogen, Leaf Phosphorus, and Specific Leaf Area: A Meta‐Analysis and Modeling Study.” Ecology and Evolution 4, no. 16: 3218–3235. 10.1002/ece3.1173. 25473475 PMC4222209

[gcb70841-bib-0051] Wolf, A. , W. R. L. Anderegg , and S. W. Pacala . 2016. “Optimal Stomatal Behavior With Competition for Water and Risk of Hydraulic Impairment.” Proceedings of the National Academy of Sciences of the United States of America 113, no. 46: E7222–E7230. 10.1073/pnas.1615144113. 27799540 PMC5135368

[gcb70841-bib-0057] Wright, J. , and M. Detto . 2026. AmeriFlux PA‐Bar Barro Colorado Island [Data set]. Princeton University. 10.17190/AMF/3015322.

[gcb70841-bib-0052] Yang, X. , R. Li , A. Jablonski , et al. 2023. “Leaf Angle as a Leaf and Canopy Trait: Rejuvenating Its Role in Ecology With New Technology.” Ecology Letters 26, no. 6: 1005–1020. 10.1111/ELE.14215. 37078440

[gcb70841-bib-0053] Yi, C. 2008. “Momentum Transfer Within Canopies.” Journal of Applied Meteorology and Climatology 47, no. 1: 262–275. 10.1175/2007JAMC1667.1.

[gcb70841-bib-0054] Yin, J. , J. D. Albertson , J. R. Rigby , and A. Porporato . 2015. “Land and Atmospheric Controls on Initiation and Intensity of Moist Convection: CAPE Dynamics and LCL Crossings.” Water Resources Research 51, no. 10: 8476–8493. 10.1002/2015WR017286.

[gcb70841-bib-0055] Yuan, H. , Y. Dai , R. E. Dickinson , et al. 2017. “Reexamination and Further Development of Two‐Stream Canopy Radiative Transfer Models for Global Land Modeling.” Journal of Advances in Modeling Earth Systems 9, no. 1: 113–129. 10.1002/2016MS000773.

[gcb70841-bib-0056] Zhou, Y. , N. Kitudom , S. Fauset , et al. 2023. “Leaf Thermal Regulation Strategies of Canopy Species Across Four Vegetation Types Along a Temperature and Precipitation Gradient.” Agricultural and Forest Meteorology 343: 109766. 10.1016/j.agrformet.2023.109766.

