# Supplementary material for: Atmospheric Boundary Layer Control on Forest Thermal Properties
Source: Glob Chang Biol. 2026 Apr 7;32(4):e70841. doi: 10.1111/gcb.70841 (PMC13058410; doi:10.1111/gcb.70841)
Supplement: Supplementary file 1 — Figure S1: Potential temperature and specific humidity profiles obtained from morning atmospheric. Figure S2: Mean Diurnal Cycle of PBL height obtained from ERAS5 Reanalysis hourly gridded product (0.25°). Figure S3: Comparison between ecosystem fluxes and 𝑇 𝑐an obtained from FORCE model simulations and the emulator. Figure S4: Comparison between observed and predicted values of the four variables used to calibrate. Figure S5: Diurnal variation of basic atmospheric forcings used to drive FORCE model. Data are obtained. [file GCB-32-e70841-s002.pdf]

Supporting information to the paper

Atmospheric boundary layer control on forest thermal properties

Matteo Detto, Christopher Still and Amilcare Porporato

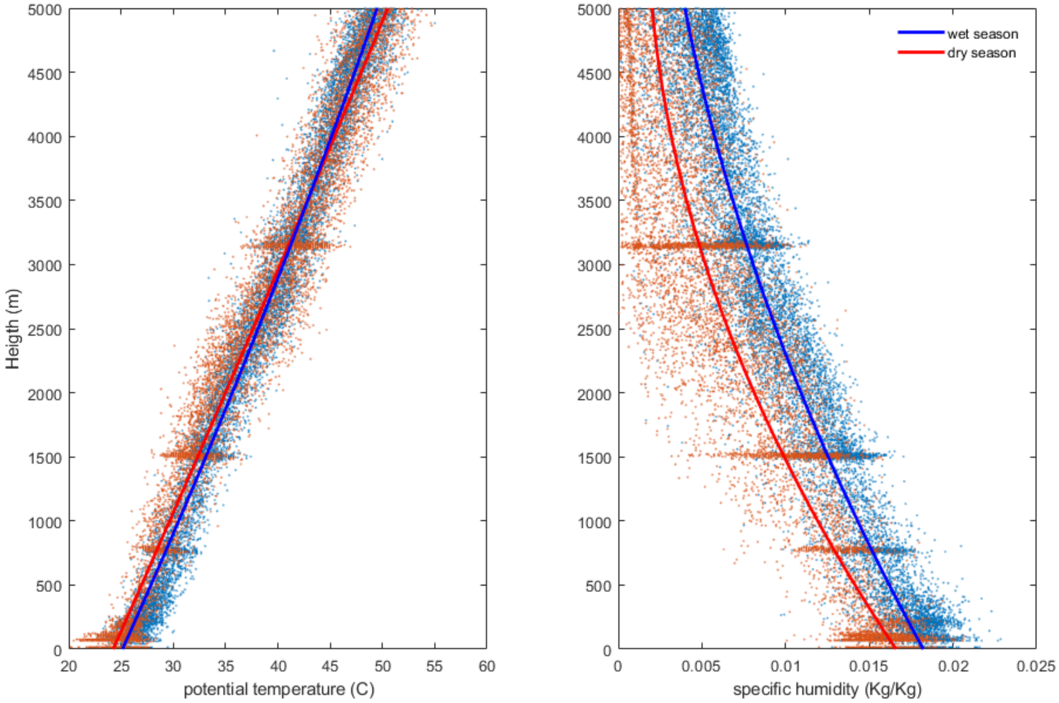

Figure S1. Potential temperature and specific humidity profiles obtained from morning atmospheric sounding at Albrook Airport for the period 1987–2001. Data are divided into wet and dry seasons. (source: https://weather.uwyo.edu/upperair/sounding.shtml). Fitted lines are shown.

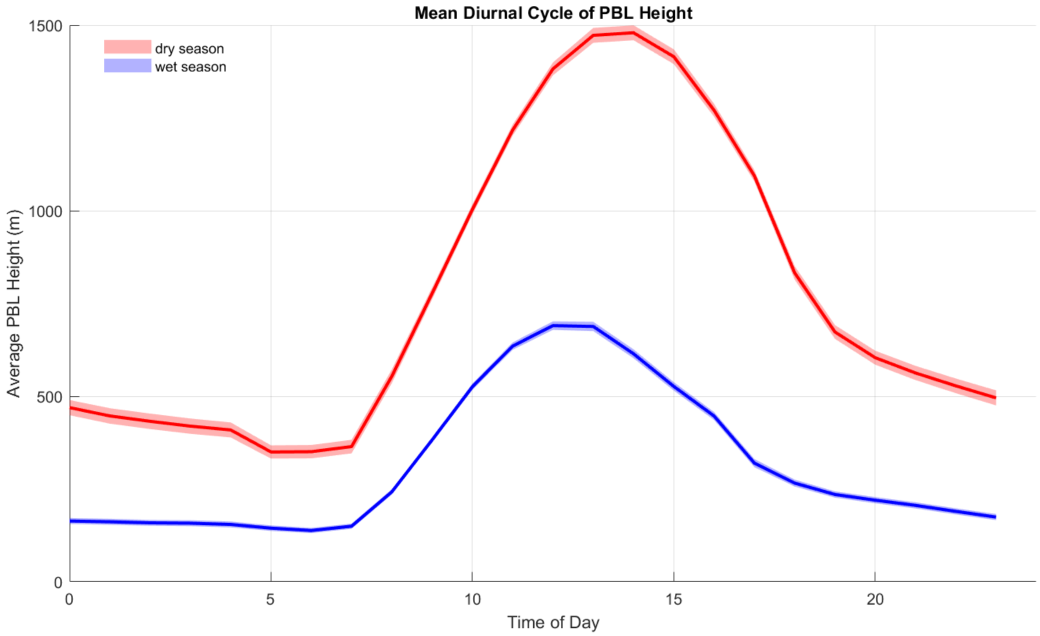

Figure S2. Mean Diurnal Cycle of PBL height obtained from ERAS5 Reanalysis hourly gridded product (0.25 degree) for year 2024 (source: https://cds.climate.copernicus.eu/datasets/reanalysis-era5-single-levels?tab=download).

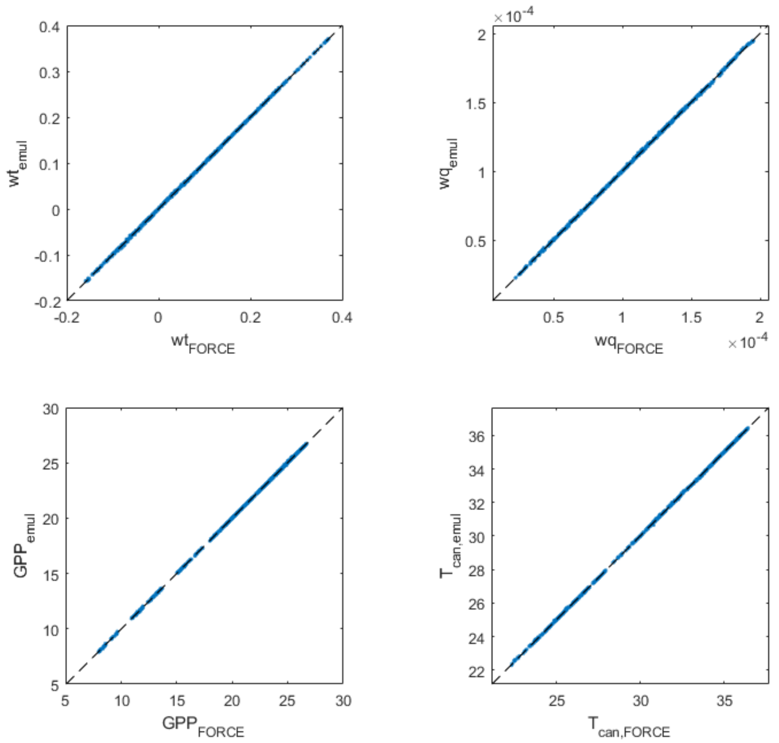

Figure S3. Comparison between ecosystem fluxes and T_{can} obtained from FORCE model simulations and the emulator. The emulator is created by training an Artificial Neural Network (ANN) by simulating the FORCE model over a wide range of climatic conditions obtained from observations conducted on Barro Colorado Island (Detto and Pacala, 2021).

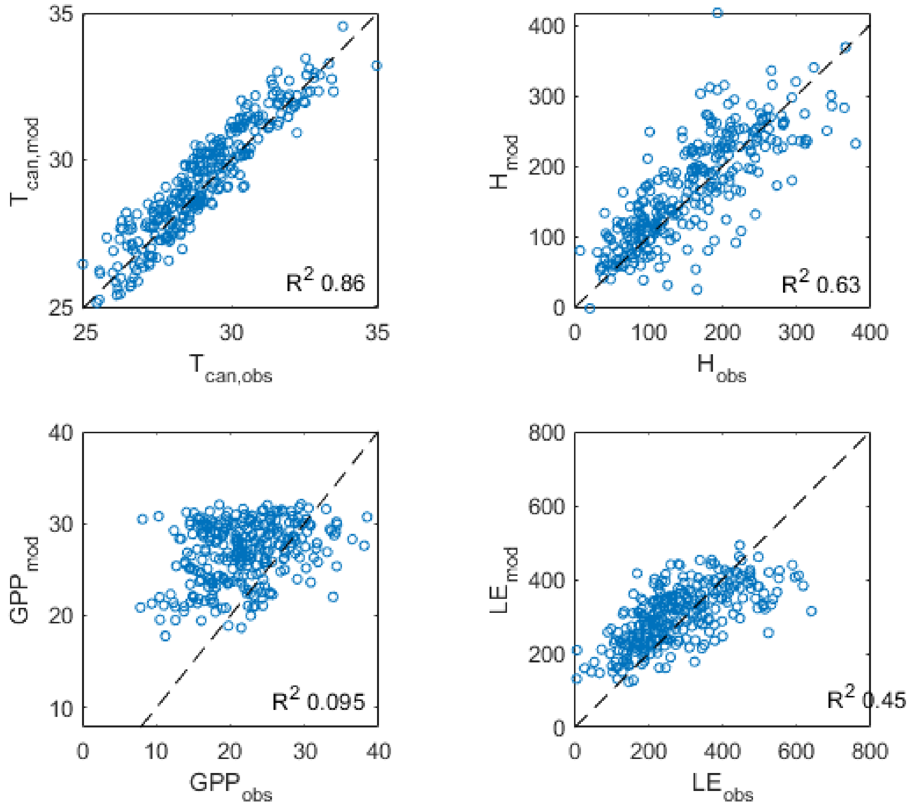

Figure S4. Comparison between observed and predicted values of the four variables used to calibrate the model parameters. R^2 are shown. observations conducted on Barro Colorado Island (Detto and Pacala, 2021).

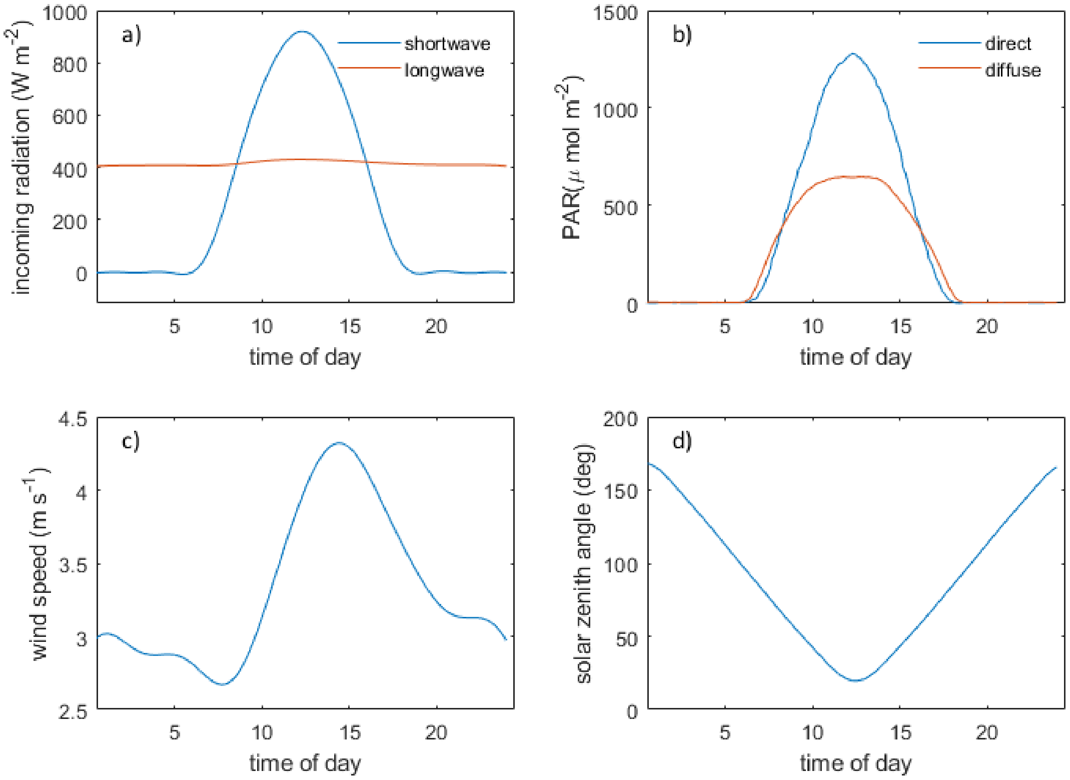

Figure S5. Diurnal variation of basic atmospheric forcings used to drive FORCE model. Data are obtained from a microclimatic tower located on Barro Colorado Island (Panama) and averaged during the dry season (mid Dec to mid-April) and during clear sky conditions (daily integrated diffuse radiation < 50% of total).
