# Supplementary material for: Atmospheric Boundary Layer Control on Forest Thermal Properties
Source: Glob Chang Biol. 2026 Apr 7;32(4):e70841. doi: 10.1111/gcb.70841 (PMC13058410; doi:10.1111/gcb.70841)
Supplement: Supplementary file 2 — Data S1: The Forest Canopy Exchange Model—FORCE‐1.0. [file GCB-32-e70841-s003.pdf]

The Forest Canopy Exchange Model – FORCE-1.0

The Forest Canopy Exchange model (FORCE) version 1.0 is a multi-layer model to simulate energy, water and carbon fluxes from forest ecosystems developed by Matteo Detto at the High Meadows Environmental Institute of Princeton University. This document describes model development and its parametrization.

Model description

The model is structured into four components

1. (I) *Radiative transfer model through the canopy*
2. (II) *Photosynthesis*
3. (III) *Leaf energy budget*
4. (IV) *Coupled hydraulic model and Optimal stomatal conductance*
5. *Vertical parametrization*
6. *Table 1 – list of variables used in the radiative transfer model*
7. *Table 2 – list of variables used in the photosynthesis model*
8. *Table 3 – list of variables used in the leaf energy model*
9. *Table 4 – list of variables used in the stomatal model*
10. *References*

1. *Radiative transfer model*

Penetration, scatter and absorption of direct (S) and diffuse ($D^{\downarrow}$ and $D^{\uparrow}$) of light throughout the canopy in the visible (VIS, 300-700 nm), near-infrared (NIR, 700-2500 nm) and thermal bands (TIR, 2.5-50 μm) are modelled with a two-stream approximation, where ↓indicates downward radiation and ↑upward (Fig. 1). For VIS and NIR bands, the two-stream model can be expressed by the following system of linear differential equations (Meador & Weaver 1980):

$$\frac{dS}{dx} = -kS \qquad (1.1a)$$

$$\frac{dD^{\downarrow}}{dx} = -(\alpha+\beta)D^{\downarrow} + \beta D^{\uparrow} + \sigma' S \qquad (1.1b)$$

$$\frac{dD^{\uparrow}}{dx} = (\alpha+\beta)D^{\uparrow} - \beta D^{\downarrow} - \sigma S \qquad (1.1c)$$

where x is the vertical coordinate expressed as cumulative leaf area from the top of the canopy ($x = 0$) to the ground ($x = \mathrm{LAI}$), α and γ are the absorption and backscatter coefficients for diffuse radiations, σ' and σ are backward and forward scatter coefficients for direct radiation, and k is the extinction coefficient of direct radiation.

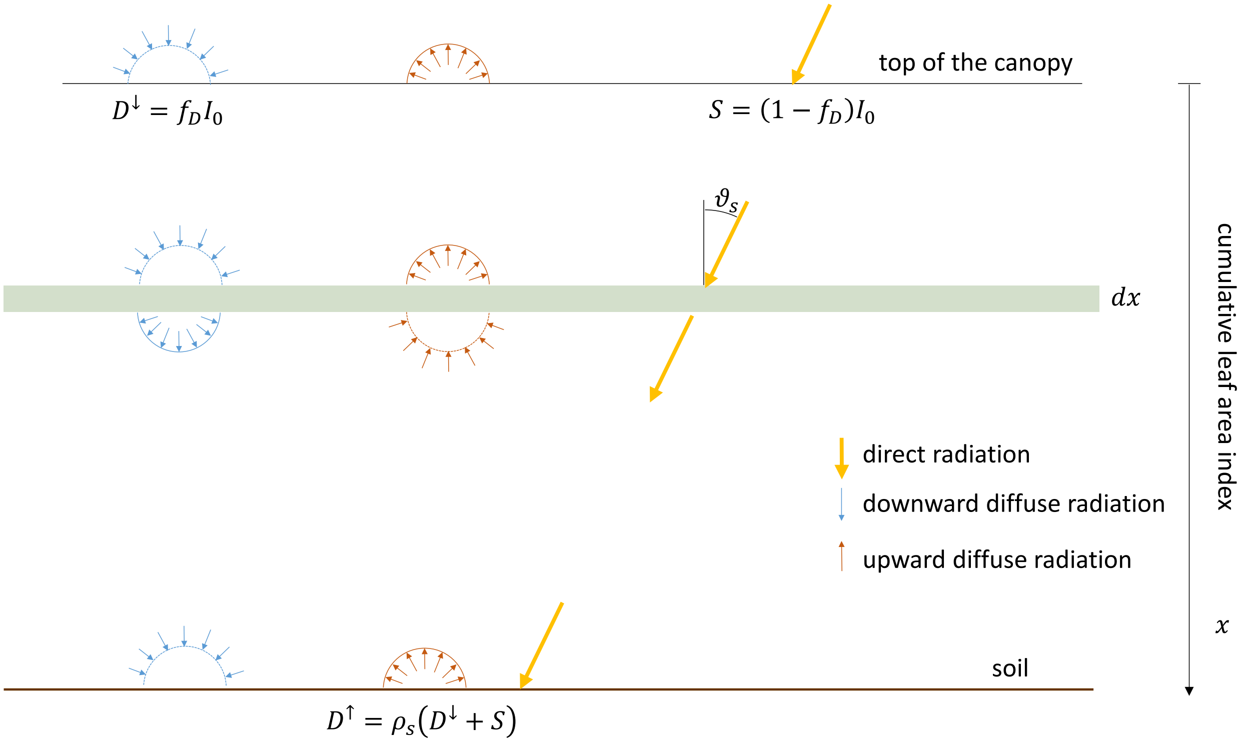

Figure 1. Schematic illustration of the two-stream radiative transfer model. The different radiations are represented with different colors, blue for downward diffuse, red for upward diffuse and yellow for direct. The diffuse radiations are assumed to be isotropic in the upper and lower hemispheres. The direct radiation is in the direction of the solar zenith angle (ϑ_s). The cartoon shows a hypothetical layer with infinitesimal depth dx with all the incoming and outgoing fluxes. Boundary conditions at the top of the canopy and forest floor are also defined.

The coefficients parametrization depends on canopy elements optical properties (reflectance, ρ and transmittance, τ), geometrical properties (angle distribution function, g), and a canopy structure (clumping index ζ). These parameters can vary with depth into the canopy.

The extinction coefficient for direct radiation is the inverse of the optical depth in the direction of the beam ϑ and is computed as:

$$k = \frac{G(\vartheta)\zeta(\vartheta)}{cos(\vartheta)}$$

where the so-called Ross G-function represents the mean area projected in the direction of the beam. and $\zeta(\vartheta)$ is the clumping index. Assuming azimuthal isotropy, the G-function in the zenith direction ϑ is computed as (Ross 1981):

$$\frac{G(\vartheta)}{cos(\vartheta)} = \int_0^{\pi/2} g(\vartheta_L)\, cos(\vartheta_L) d\vartheta_L + 2\int_{\pi/2-\vartheta}^{\pi/2} g(\vartheta_L)\, cos(\vartheta_L) \frac{\sqrt{tan(\vartheta)^2\, tan(\vartheta_L)^2 - 1} - sec^{-1}[tan(\vartheta)\, tan(\vartheta_L)]}{\pi} d\vartheta_L \quad (1.2)$$

where $g(\vartheta_L)$ is the leaf angle distribution ($\int_0^{\pi/2} g(\vartheta_L) d\vartheta_L = 1$). The absorption and backscatter coefficients for diffuse radiations are given by (Yuan *et al.* 2017):

$$\alpha = \frac{1-\omega}{\bar{\mu}} \quad (1.3a)$$

$$\beta = \frac{1}{2}\frac{\omega + J\delta}{\bar{\mu}} \quad (1.3b)$$

where $\omega = \rho + \tau$ and $\delta = \rho - \tau$ and $\bar{\mu}$ is the average optical depth for diffuse radiation computed as:

$$\bar{\mu} = \int_0^1 \frac{\mu}{G(\mu)\zeta(\mu)} d\mu \quad (1.4)$$

with $\mu = \cos(\vartheta)$ and

$$J = \int_0^{\pi/2} g(\vartheta)\cos(\vartheta)^2\, d\vartheta \quad (1.5)$$

The backward and forward scattering for direct radiation are given by (Pinty *et al.* 2006):

$$\sigma' = \frac{1}{2}(\omega k + J\delta) \quad (1.6a)$$

$$\sigma = \omega k - \sigma' \quad (1.6b)$$

The system is subjected to the following boundary conditions

$$S = (1 - f_D)I_0 \quad \text{at } x = 0 \quad (1.7a)$$

$$D^{\downarrow} = f_D I_0 \quad \text{at } x = 0 \quad (1.7b)$$

$$D^{\uparrow} = \rho_s(D^{\downarrow} + S) \quad \text{at } x = LAI \quad (1.7c)$$

where ρ_s is the Lambertian soil reflectance, f_D is the fraction of incoming diffuse radiation and I_0 is the total incoming radiation (for VIS or NIR).

For TIR, there is no direct component, and the diffuse components must be modified to include radiation emitted by leaf (Yang *et al.* 2020):

$$\frac{dD^{\downarrow}}{dx} = -(\alpha + \gamma)D^{\downarrow} + \gamma D^{\uparrow} + \varepsilon\sigma T_L^4 \tag{1.8a}$$

$$\frac{dD^{\uparrow}}{dx} = \quad (\alpha + \gamma)D^{\uparrow} - \gamma D^{\downarrow} - \varepsilon\sigma' T_L^4 \tag{1.8b}$$

where T_L is leaf temperature in Kelvin. The boundary conditions are given by:

$$D^{\downarrow} = L_0 \qquad \text{at } x = 0 \tag{1.9a}$$

$$D^{\uparrow} = \rho_S D^{\downarrow} + \sigma\varepsilon_S T_S^4 \qquad \text{at } x = LAI \tag{1.9b}$$

where T_s is the soil temperature and L_0 is the longwave incoming radiation.

The system of differential equations (1.1) and (1.3), with boundary conditions (1.2) and (1.4) and the parametrization given in Table 1 is solved numerically using a boundary value problem algorithm, *bvp5c.m*, in MATLAB (R2019a). The integrals are computed numerically with the function *integral.m*.

Absorbed radiation

The calculations of leaf temperature and photosynthesis are nonlinear functions of absorbed radiation. Because leaves within the same canopy layer experience different radiation conditions depending on whether they are sunlit or shaded, as well as their orientation relative to the sun, absorbed radiation must be computed separately for each group (Norman 1980; De Pury & Farquhar 1997).

Sunlit leaves receive both direct solar radiation from the direction r_S and diffuse radiation from the lower and upper hemispheres. Since the diffuse radiation components are assumed to be isotropic and leaf optical properties are equal for both leaf sides, the absorbance of diffuse radiation is independent of leaf orientation. In contrast, absorbed direct radiation depends on the leaf orientation (r_L) in relation to sun direction (r_S). The absorbance per unit of leaf area is computed as

$$Q_{sun}(r_L, r_S) = \alpha \frac{(1-f_D)I_0}{\cos(\vartheta_S)} |r_L \cdot r_S| + \alpha \frac{D^{\downarrow}+D^{\uparrow}}{\bar{\mu}} \tag{1.10}$$

where the dot product $r_L \cdot r_S$ indicates the cosine of the angle between the direction of the sun and the normal to the leaf (Ross 1981).

Thus, sunlit leaves must be classified based on the magnitude of $|r_L \cdot r_S|$ (Norman 1980). The probability of a leaf having a specific orientation relative to the solar direction is numerically determined for each depth. In summary, an azimuth-zenith angle grid (ϕ, ϑ) is first generated, with uniform spacing along the azimuthal axis and spacing along the zenith axis adjusted according to the leaf angle distribution using the inverse of the Beta cumulative distribution function. The absolute value of the cosine of the angle between the sun and leaf direction is then computed at each grid point using the law of cosines:

$$|r \cdot r_S| = |\cos(\vartheta_S)\cos(\vartheta) + \sin(\vartheta_S)\sin(\vartheta)\cos(\phi)| \tag{1.11}$$

$|r \cdot r_S|$is divided into quantiles between 0 and 1 and the mean angle of each class is calculated from the grid. Examples of frequency histograms for different leaf angle distributions are shown in Fig. 2.

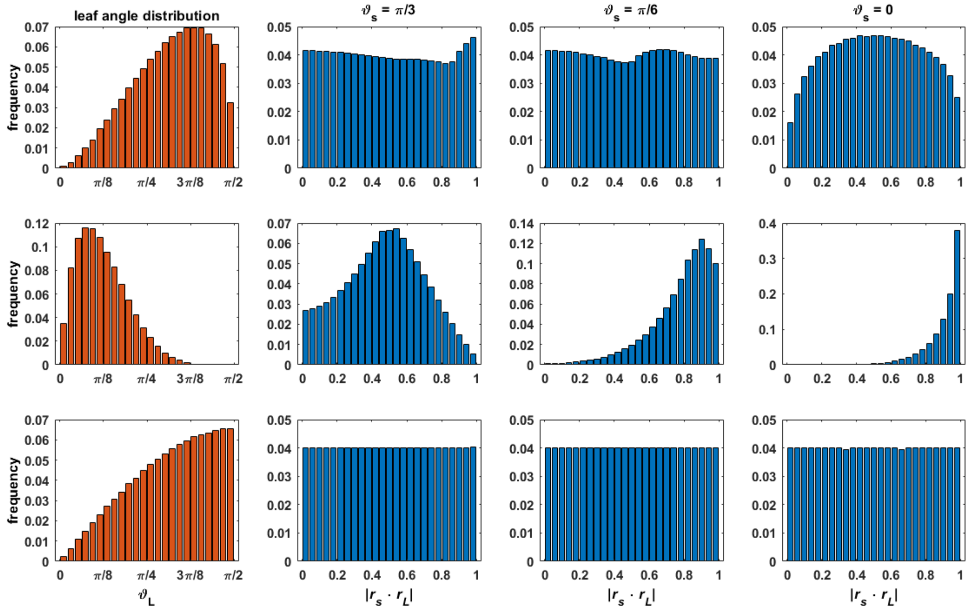

Figure 2. Frequency histograms (in blue) of $|r_L \cdot r_s|$, the absolute value of the cosine of the angle between the sunray directions and the normal to the leaf surface for different solar zenith angles and different beta leaf angle distributions (in red). **Top**) for a leaf angle distribution typical of the top of the canopy with preferentially vertically oriented leaves. **Middle**) for a distribution typical of the understory with leaves with flatter leaf angles. **Bottom**) for spherical angle distribution, the distribution of $|r_L \cdot r_s|$is uniform and independent of the sunray angle.

A shaded leaf receives only diffuse and scattered radiations, and its absorbance is equal to

$$Q_{shade} = \alpha \frac{D^{\downarrow}+D^{\uparrow}}{\bar{\mu}} \tag{1.12}$$

The probability of a leaf being sunlit is the gap fraction multiplied by the clumping index (assumed to be isotropic), i.e.:

$$p(x) = \frac{S(x)}{S(0)}\zeta \tag{1.13}$$

2. *Photosynthesis*

The instantaneous rate of net photosynthesis is derived using the classic Farquhar model via Michaelis-Menten type dependence upon CO_2 concentration in the mesophyll c_i (Farquhar *et al.* 1980):

$$A_n = min\left[V_{cmax}\frac{c_i-\Gamma_*}{k_c\left(1+\frac{O}{k_O}\right)+c_i}, J\frac{c_i-\Gamma_*}{4c_i+8\Gamma_*}\right] - R_d \tag{2.1}$$

where V_{cmax} is the maximum carboxylation rate, J is the electron transport rate, Γ_* is the CO_2 compensation point, k_c and k_o are the Michaelis-Menten constants for CO_2 and O_2, respectively, O is the oxygen concentration, R_d is the dark leaf respiration. J is a saturating function of irradiance, converging on J_{max}:

$$\theta J^2 - (\alpha_{(LL)}Q + J_{max})J + \alpha_{(LL)}QJ = 0 \tag{2.2}$$

where Q is the absorbed photosynthetically active radiation, θ is the curvature of the light response curve, and $\alpha_{(LL)}$ is the realized quantum yield of photosynthetic electron transport (Farquhar & Wong 1984).

The temperature dependence of the kinetic constants can be described by an Arrhenius function:

$$y_{i,T} = y_{i,25} e^{\frac{T-298}{298RT}\Delta H_i} \quad (2.3)$$

where $y_i = (\Gamma_*, k_c, k_o, R_d)$ and T is the temperature in Kelvin, ΔH_i is the activation energy for parameter i and R the universal gas constant (Bernacchi *et al.* 2001). For $y_i = (V_{cmax}, J_{max})$, we used a modified Arrhenius function as in Medlyn et al. (2002):

$$y_{i,T} = y_{i,25} e^{\frac{T-298}{298RT}\Delta H_i} \frac{1+e^{\frac{298\Delta S_i - H_i}{298R}}}{1+e^{\frac{T\Delta S_i - H_i}{RT}}} \quad (2.4)$$

The entropy factor ΔS is expressed a function of optimal temperature T_{opt}

$$\Delta S_i = \frac{\Delta H_i}{T_{opt,i}} + R \log\left(\frac{\Delta H_i}{H_i - \Delta H_i}\right) \quad (2.5)$$

Generally, T_{opt} for V_{cmax} is larger than T_{opt} for J_{max} (Crous *et al.* 2022). c_i is related to air concentration via Fick's law and conductance for CO2 g_c (see next section):

$$A_n = g_c(c_a - c_i) \quad (2.6)$$

We can eliminate c_i from the previous equations (2.1) and (2.6) in order to obtain an expression function only of g_c

$$A_n = a + bg_c - \sqrt{b^2 g_c^2 + cg_c + a^2} \quad (2.7)$$

where for carbon limited

$$2a = V_{cmax} - R_d$$
$$2b = K_m + c_a$$
$$2c = R_d(c_a + K_m) + V_{cmax}(K_m + 2\Gamma_* - c_a) \quad (2.8a)$$

with $k_m = k_c(1 + O/k_o)$, and for light-limited

$$2a = J/4 - R_d$$
$$2b = c_a + 2\Gamma^*$$
$$2c = R_d(c_a + 2\Gamma_*) + J(\Gamma_* - c_a/4) \quad (2.8b)$$

3. *Leaf energy budget*

Leaf temperature is computed from theory of leaf energy balance (Leuning *et al.* 1989), expressed by the following budget equation:

$$Q = 2\varepsilon\sigma T_L^4 + 2c_p g_{Ha}(T_L - T_{air}) + \lambda g_v \frac{e_L^* - e_a}{p_a} \quad (3.1)$$

The boundary layer conductance for heat, water vapor, and CO_2 are computed as a sum of force and free convection (Campbell & Norman 1998)

$$g_{Ha} = 1.4 \times 0.135\sqrt{u/d} + 0.05\left(\frac{T_L - T_{air}}{d}\right)^{0.25} \quad (3.2a)$$

$$g_{va} = 1.4 \times 0.147\sqrt{u/d} + 1.09\left(\frac{T_L - T_{air}}{d}\right)^{0.25} \quad (3.2b)$$

$$g_{ca} = 1.4 \times \underbrace{0.110\sqrt{u/d}}_{forced} + \underbrace{0.75\left(\frac{T_L - T_{air}}{d}\right)^{0.25}}_{free} \quad (3.2c)$$

where 1.4 is a conductance enhancement factor to account for objects placed in natural turbulent winds. Total conductance for water vapor and CO_2 are given by

$$g_v = \frac{g_{va} g_s}{g_{va} + g_{vs}} \quad (3.3a)$$

$$g_c = \frac{g_{ca} g_s / 1.6}{g_{ca} + g_s / 1.6} \quad (3.3b)$$

where 1.6 is a factor for accounting difference in molecular diffusivity between H_2O and CO_2. (3.1) is solved numerically using the MATLAB function *fzero*.

4. Optimal stomatal control and plant hydraulics

Optimal stomatal conductance is obtained as a maximization problem of total carbon gain, formulated as (Cowan & Farquhar 1977):

$$\max_{g_s}(A_n - \Theta) \quad (4.1)$$

where Θ is the cost of water consumption, typically a concave-up function of leaf water potential ψ_L (Wolf *et al.* 2016). ψ_L was computed from a single-element hydraulic model scaled per unit of leaf area without storage. The flow through the element is calculated by integrating Darcy law along the hydraulic path.

$$E = \int_{\psi_L}^{\tilde{\psi}_s} K_p(\psi)\,d\psi \quad (4.2)$$

where K_P is the whole element hydraulic conductance and where $\tilde{\psi}_s$ is the root-weighted average soil water potential computed as $\tilde{\psi}_s = \frac{\sum_i a_{R,i}\psi_{s,i}}{\sum_i a_{R,i}}$, with $\frac{a_{R,i}}{\sum_i a_{R,i}}$ representing the proportion of root absorbing area in the ith-soil layer, given by an exponential vertical root distribution (Jackson *et al.* 1996). $K_p(\psi)$ attains a maximum value K_{max} for $\psi = 0$, and decreases for negative plant water potentials following a two-parameters sigmoidal vulnerability curve, defined by the water potential corresponding to a 50% loss in conductivity, p_{50}, and the slope at the inflection point s:

$$K_p(\psi) = K_{max}\frac{e^{s(\psi - p_{50})}}{e^{s(\psi - p_{50})} + 1} \quad (4.3)$$

(4.2) can be expressed in terms of matrix potentials as:

$$E = \Phi_S - \Phi_L \quad (4.4)$$

where the matrix potential is defined as

$$\Phi_x = \int_{-\infty}^{\psi_x} K_p(\psi)\,d\psi = \frac{K_{max}}{s}\log\left(e^{s(\psi_x - p_{50})} + 1\right) \quad (4.5)$$

We assume that the cost Θ is a concave-up function of leaf water potential ψ_L (Wolf *et al.* 2016), with a vertical asymptote at the leaf turgor loss point, which is the water potential at which the leaf loses its functionality, to guarantee that leaf water potential remains within the turgor loss point threshold. For practical reasons, we also express Θ in terms of matrix potentials as:

$$\Theta = c_w \frac{\Phi_L}{\Phi_L - \Phi_{\pi_{tlp}}} \tag{4.6}$$

where c_w is an empirical parameter and π_{tlp} is the turgor loss point. Using (4.4), (4.6) can be expressed explicitly as a function of the evaporation rate E as:

$$\Theta = c_w \frac{\Phi_S - E}{\Phi_S - E - \Phi_{\pi_{tlp}}} \tag{4.7}$$

Given $E = g_v \frac{e_L^* - e_a}{p_a}$, Eq. (4.7), (2.1) and (3.1) constitute a closed system that can be solved numerically to search for the optimal g_s that maximizes (4.1). The search is restricted between g_{min} (cuticular conductance) and g_{max}.

5. *Vertical parametrization*

The model is solved on a regular grid along the vertical axis, x. We assume air temperature, T_{air}, and water vapor pressure, e_{air} constant along the canopy, while wind speed U varies exponentially

$$U = U^{(top)} e^{-\frac{1}{2}x} \tag{5.1}$$

where $U^{(top)}$ is the wind speed at the canopy top (Yi 2008).

At reference temperature (25°C), the maximum electron transport rate and dark respiration are considered proportional to $V_{cmax,25}$ (Monson & Baldocchi 2014). Based on vertical data collected at PNM by Lamour et al. (2023), indicating that leaf dark respiration declines faster than $V_{cmax,25}$ with canopy depth, while $J_{max.25}$:$V_{cmax,25}$ remains constant, $V_{cmax,25}$, $J_{max,25}$ and $R_{d,25}$ at each canopy depth, x, were parametrized as:

$$V_{cmax,25} = V_{cmax,25}^{(top)}(1 - 0.075x) \tag{5.2a}$$

$$J_{max,\,25} = 1.96 V_{cmax,25} \tag{5.2b}$$

$$R_{d,25} = 0.015 V_{cmax,25}^{(top)}(1 - 0.11x) \tag{5.2c}$$

The canopy is assumed to be composed of leaves and woody elements with a constant fraction, f_w. Woody elements are parametrized as leaves but with different optical properties, in particular no transmittance, and erectophile angle distribution. The angle distribution and optical properties for the composite canopy are obtained as a weighted averages of leaves and woody elements as:

$$g(\vartheta_L) = (1 - f_w) g_L(\vartheta_L) + g_w(\vartheta_L)$$

$$\rho = (1 - f_w)\rho_L + \rho_w$$

$$\tau = (1 - f_w)\tau_L$$

Mean and standard deviation of the leaf angle distribution were parametrized with data collected from several towers on BCI (Detto *et al.* 2015), indicating more vertical distribution at the canopy top and flatter in the understory. These variables were also assumed to vary linearly with canopy depth as:

$$\mu_L = \mu_L^{(top)}(1 - 0.105x) \tag{5.3a}$$

$$\sigma_L = \sigma_L^{(top)}(1 - 0.058x) \tag{5.3b}$$

μ_L and σ_L are related to the parameters of the Beta distribution

$$g_L(\vartheta_L) = \frac{2}{\pi}\frac{1}{B(\mu,\nu)}\left(1 - \frac{2\vartheta_L}{\pi}\right)^{\mu-1}\left(\frac{2\vartheta_L}{\pi}\right)^{\nu-1} \tag{5.4}$$

by the following relationships:

$$\nu = \bar{t}\left(\frac{s_0^2}{s_t^2} - 1\right)$$

$$\mu = (1 - \bar{t})\left(\frac{s_0^2}{s_t^2} - 1\right)$$

where $\bar{t} = \frac{\mu_\vartheta}{\pi/2}$, $s_t^2 = \left(\frac{\sigma_\vartheta}{\pi/2}\right)^2$ and $s_0^2 = \bar{t}(1 - \bar{t})$ and $B(\mu, \nu)$ is the Beta function. For woody elements we used an erectophile distribution given by

$$g_w(\vartheta_L) = \frac{2}{\pi}(1 - \cos(1 - 2\vartheta_L))$$

All other parameters, including leaf optical properties and hydraulic parameters are constant within the canopy. The energy balance is computes separately for leaves and woody elements, assuming the wood does not transpire.

A MATLAB code is provided at https://github.com/mdetto/FORCE-1.0.

Table 1. Symbolic notation, definition and parametrization used for the radiative transfer model

Symbol	Description	Unit	Parametrization
ρ ,τ	leaf reflectance and transmittance	$-$	$user\ defined\ (u.d.)$
ρ_s	soil reflectance	$-$	$u.d.$
ϑ_s	solar zenith angle	rad	forcing
ϑ_L	angle between the normal direction of the leaf and the vertical axis	rad	
$g_L(\vartheta_L)$	leaf angle distribution	rad^{-1}	$\frac{2}{\pi}\frac{1}{B(\mu,\nu)}\left(1-\frac{2\vartheta_L}{\pi}\right)^{\mu-1}\left(\frac{2\vartheta_L}{\pi}\right)^{\nu-1}$
μ_ϑ	mean leaf angle	rad	$\mu_\vartheta^{(top)}(1-1.05x)$
σ_ϑ	standard deviation of leaf angle	rad	$\sigma_\vartheta^{(top)}(1-0.058x)$
$G(\vartheta)$	mean projection of leaf area in the direction ϑ	$-$	Eq. (1.5)
J	J-function	$-$	$\int_0^{\pi/2} g(\vartheta_L)\,cos(\vartheta_L)^2 d\vartheta_L$
I_0	total incoming irradiance	$\mathrm{W\ m^{-2}}$	forcing
$D^{\downarrow}, D^{\uparrow}$	downward and upward diffuse fluxes	$\mathrm{W\ m^{-2}}$	
S	direct solar radiation	$\mathrm{W\ m^{-2}}$	
f_D	fraction of incoming diffuse radiation	$-$	forcing
ω	single scattering albedo for an individual leaf	$-$	$\rho+\tau$
ζ	clumping index	$-$	$u.d.$
x	cumulative leaf area index	$\mathrm{m^2 m^{-2}}$	
α	absorption coefficient	$-$	$1-\omega$
γ	backward scattering for diffuse radiation	$-$	$\frac{1}{2}(\omega+J\delta)$
k	extinction coefficient for direct radiation	$-$	$G(\vartheta_s)/\cos(\vartheta_s)$
σ	backward scattering for direct radiation	$-$	$\frac{1}{2}(\omega k+J\delta)$
σ'	forward scattering for direct radiation	$-$	$\omega k-\sigma$
T_L	leaf temperature	K	
T_s	soil temperature	K	
ε, ε_s	leaf and soil emissivity	$-$	$u.d.$
r_L	direction of leaf normal	$-$	
r_s	direction of the sun	$-$	forcing
f_w	fraction of woody area	$-$	$u.d.$

all scattering and extinction coefficients are given per unit of leaf area

$B(\mu,\nu)$ is the beta function

Table 2. Symbolic notation, definition and parametrization used for the photosynthesis model

**Symbol**	**Description**	**Unit**	**Parametrization**
$V_{cmax,25}$	maximum carboxylation velocity at 25°C	μmol m^{2} s^{-1}	$V_{cmax,25}^{(top)}(1-0.075x)$
$J_{max,25}$	maximum electron transport rate at 25°C	μmol m^{2} s^{-1}	$1.96V_{cmax,25}$
$R_{d,25}$	leaf dark respiration at 25°C	μmol m^{2} s^{-1}	$0.015\times V_{cmax,25}(1-0.11x)$
J	electron transport rate	μmol m^{2} s^{-1}	Eq. (2.2)
$\Gamma_{*,25}$	CO_2 compensation point	μmol mol^{-1}	42.75
$k_{c,25}$	Michaelis-Menten constant for CO_2	μmol mol^{-1}	404.9
$k_{o,25}$	Michaelis-Menten constant for O_2	mmol mol^{-1}	278.4
c_a	ambient CO_2 concentration	μmol mol^{-1}	forcing
c_i	intercellular CO_2 concentration	μmol mol^{-1}	
O	oxygen concentration	mmol mol^{-1}	210
A_n	net photosynthesis	μmol m^{2} s^{-1}	Eq. (2.1)
θ	curvature of the light response curve	–	0.7
$\alpha_{(LL)}$	quantum yield of electron transport	–	0.36
Q	absorbed PAR	μmol m^{2} s^{-1}	$Q=4I_{(VIS)}$
ΔH	activation energy	J mol^{-1}	$\Delta H_{k_c}=7043, \Delta H_{k_o}=36380, \Delta H_{V_{cmax}}=66560$ $\Delta H_{\Gamma_*}=37830, \Delta H_{R_d}=46390, \Delta H_{J_{max}}=39650$
H	rate of decrease of above the optimum	J mol^{-1}	$H_{V_{cmax}}=\Delta H_{J_{max}}=200\times10^3$
T_{opt}	optimal temperature	K	$T_{opt,V_{cmax}}=42$, $T_{opt,J_{max}}=38$
ΔS_y	entropy factor for variable y	J mol^{-1}	Eq. (2.5)
g_c	leaf conductance for CO_2	mol m^{2} s^{-1}	
R	universal gas constant	J mol^{-1} K^{-1}	8.414

Table 3. Symbolic notation, definition and parametrization used for the leaf energy budget

Symbol	Description	Unit	Parametrization
T_L	leaf and air temperature	K	Eq. (3.1)
T_a	leaf and air temperature	K	forcing
e_a	air water vapor pressure	Pa	forcing
e_L^*	leaf-saturated water vapor pressure	Pa	$610.94\ e^{\frac{17.625\times T_L}{T_L+243.04}}$
p_a	air pressure	Pa	forcing
σ	Stephan-Bolzman constant	$\mathrm{Wm^{-2}K^{-4}}$	5.6703×10^{-8}
ε	leaf emissivity	$-$	$u.d.(0.97)$
c_p	air specific heat capacity	$\mathrm{J\ mol^{-1}}K^{-1}$	29.3
λ	latent heat of evaporation	$\mathrm{J\ mol^{-1}}$	43.9
g_{Ha}	heat boundary layer conductance	$\mathrm{mol\ m^{-2}}s^{-1}$	$1.4\times0.135\sqrt{U/d}+0.05\left(\frac{T_L-T_{air}}{d}\right)^{0.25}$
g_{va}	H_2O boundary layer conductance	$\mathrm{mol\ m^{-2}}s^{-1}$	$1.4\times0.147\sqrt{U/d}+1.09\left(\frac{T_L-T_{air}}{d}\right)^{0.25}$
g_{ca}	CO_2 boundary layer conductance	$\mathrm{mol\ m^{-2}}s^{-1}$	$1.4\times0.110\sqrt{U/d}+0.75\left(\frac{T_L-T_{air}}{d}\right)^{0.25}$
g_s	stomatal conductance to H_2O	$\mathrm{mol\ m^{-2}}s^{-1}$	computed by optimization
g_v	total conductance to H_2O	$\mathrm{mol\ m^{-2}}s^{-1}$	$\frac{g_{va}g_s}{g_{va}+g_s}$
g_c	total conductance to CO_2	$\mathrm{mol\ m^{-2}}s^{-1}$	$\frac{g_{ca}g_s}{1.6g_{ca}+g_s}$
R_{abs}	radiation absorbed by the leaf	$\mathrm{Wm^{-2}}$	forcing
d	characteristic leaf size	m	0.15
$u(x)$	wind speed at the canopy depth x	$\mathrm{m\ s^{-1}}$	$u_0e^{-\frac{1}{2}x}$
x	cumulative LAI	$\mathrm{m^2\ m^{-2}}$	

Table 4. Symbolic notation, definition and parametrization used in stomatal optimization

Symbol	Description	Unit	Parametrization
Θ	cost function	$\mu\text{mol m}^{-2}\text{ s}^{-1}$	$c_w \frac{\Phi_L}{\Phi_L - \Phi_{\pi_{tlp}}}$
c_w	empirical parameters of cost function	$\mu\text{mol m}^{-2}\text{ s}^{-1}$	$u.d$ (5)
ψ_L	leaf water potentials	Mpa	
$\psi_{S,i}$	soil water potential in the layer i	Mpa	forcing
$\tilde{\psi}_S$	root weighted average soil water potential	Mpa	$\frac{\Sigma_i a_{R,i}\psi_{s,i}}{a_{R,tot}}$
K_p	whole-plant hydraulic conductance per unit of leaf area	$\text{mol m}^{-2}\text{ s}^{-1}\text{Mpa}^{-1}$	$K_{max}\frac{e^{\alpha(\psi - p_{50})}}{e^{\alpha(\psi-p_{50})}+1}$
K_{max}	maximum hydraulic conductivity	$\text{mol m}^{-2}\text{ s}^{-1}\text{Mpa}^{-1}$	$u.d$ (4)
s	slope at the inflection point of K_p	Mpa^{-1}	$u.d$ (1)
p_{50}	water potential for a 50% loss in conductivity	Mpa	$u.d.(-1.5)$
π_{tlp}	turgor loss point	Mpa	$u.d.(-2)$
g_{min}	minimum (cuticular) stomatal conductance	$\text{mol m}^{-2}\text{ s}^{-1}$	$u.d.(0.01)$
g_{max}	maximum stomatal conductance	$\text{mol m}^{-2}\text{ s}^{-1}$	$u.d.(0.5)$

References

Bernacchi, C.J., Singsaas, E.L., Portis Jr, A.R., Pimentel, C. & Long, S.P. (2001). Improved temperature response functions for models of Rubisco-limited photosynthesis. *Plant Cell Environ*, 24, 253–259.

Campbell, G. & Norman, J.M. (1998). An Introduction to Environmental Biophysics. *J Environ Qual*, Heidelberg Science Library, 6, 474–474.

Cowan, I.R. & Farquhar, G.D. (1977). Stomatal function in relation to leaf metabolism and environment. *Symp Soc Exp Biol*, 31, 471–505.

Crous, K.Y., Uddling, J. & De Kauwe, M.G. (2022). Temperature responses of photosynthesis and respiration in evergreen trees from boreal to tropical latitudes. *New Phytologist*, 234, 353–374.

Detto, M., Asner, G.P., Muller-landau, H.C. & Sonnentag, O. (2015). Spatial variability in tropical forest leaf area density from multireturn lidar and modeling. *Journal of Geophysical Research-Biogeosciences*, 294–309.

Farquhar, G., Caemmerer, S. & Berry, J. (1980). A biochemical model of photosynthetic CO 2 assimilation in leaves of C 3 species. *Planta*, 149, 78–90.

Farquhar, G. & Wong, S. (1984). An Empirical Model of Stomatal Conductance. *Functional Plant Biology*, 11, 191–210.

Jackson, R.B., Canadell, J., Ehleringer, J.R., Mooney, H.A., Sala, O.E. & Schulze, E.D. (1996). A global analysis of root distributions for terrestrial biomes. *Oecologia*, 108, 389–411.

Lamour, J., Davidson, K.J., Ely, K.S., Le Moguédec, G., Anderson, J.A., Li, Q., *et al.* (2023). The effect of the vertical gradients of photosynthetic parameters on the CO2 assimilation and transpiration of a Panamanian tropical forest. *New Phytologist*, 238, 2345–2362.

Leuning, A.R., Grace, J., Monteith, J.L., Milford, J.R., Unsworth, M.H. & Fowler, D. (1989). Leaf Energy Balances : Developments and Applications. *Philos Trans R Soc Lond B Biol Sci*, 324.

Meador, W.E. & Weaver, W.R. (1980). Two-stream approximation to radiative transfer in planetary atmospheres: a unified description of existing methods and a new improvement. *J Atmos Sci*, 37, 630–643.

Medlyn, B.E., Dreyer, E., Ellsworth, D., Forstreuter, M., Harley, P.C., Kirschbaum, M.U.F., *et al.* (2002). Temperature response of parameters of a biochemically based model of photosynthesis. II. A review of experimental data. *Plant Cell Environ*, 25, 1167–1179.

Monson, R. & Baldocchi, D. (2014). *Terrestrial Biosphere-Atmosphere Fluxes*. Cambridge University Press.

Norman, J.M. (1980). Interfacing leaf and canopy light interception models. In: *Predicting Photosynthesis for Ecosystem Models*. CRC Press, Boca Raton, pp. 49–68.

Pinty, B., Lavergne, T., Dickinson, R.E., Widlowski, J.L., Gobron, N. & Verstraete, M.M. (2006). Simplifying the interaction of land surfaces with radiation for relating remote sensing products to climate models. *Journal of Geophysical Research Atmospheres*, 111, 1–20.

De Pury, D.G.G. & Farquhar, G.D. (1997). Simple scaling of photosynthesis from leaves to canopies without the errors of big-leaf models. *Plant Cell Environ*, 20, 537–557.

Ross, I. (1981). *The radiation regime and architecture of plant stands*. Dr W Junk Publisher, The Hague.

Wolf, A., Anderegg, W.R.L. & Pacala, S.W. (2016). Optimal stomatal behavior with competition for water and risk of hydraulic impairment. *Proc Natl Acad Sci U S A*, 113, E7222–E7230.

Yang, P., Verhoef, W. & van der Tol, C. (2020). Unified four-stream radiative transfer theory in the optical-thermal domain with consideration of fluorescence for multi-layer vegetation canopies. *Remote Sens (Basel)*, 12, 1–19.

Yi, C. (2008). Momentum transfer within canopies. *J Appl Meteorol Climatol*, 47, 262–275.

Yuan, H., Dai, Y., Dickinson, R.E., Pinty, B., Shangguan, W., Zhang, S., *et al.* (2017). Reexamination and further development of two-stream canopy radiative transfer models for global land modeling. *J Adv Model Earth Syst*, 9, 113–129.
