# Supplementary material for: Atmospheric Boundary Layer Control on Forest Thermal Properties
Source: Glob Chang Biol. 2026 Apr 7;32(4):e70841. doi: 10.1111/gcb.70841 (PMC13058410; doi:10.1111/gcb.70841)
Supplement: Supplementary file 3 — Appendix A. ABL model parametrization. [file GCB-32-e70841-s001.docx]

**Appendix A. ABL model parametrization**

In order to solve the ABL growth model and coupling with surface fluxes (Eqs. 5 and 6), we need to parametrize entrainment velocity $w_{e}$, entrainment fluxes at the ABL top ${\overline{w^{'}\theta^{'}}}_{e}$ and ${\overline{w^{'}q^{'}}}_{e}$ and the aerodynamic resistance in the surface layer.

The entrainment fluxes are computed as

$${\overline{w^{'}\theta^{'}}}_{e}=w_{e}\Delta\theta$$

$${\overline{w^{'}q^{'}}}_{e}=w_{e}\Delta q$$

where $\Delta\theta$ and $\Delta q$ are the potential temperature and humidity jumps at the ABL top (Fig. A1):

$$\Delta\theta=\theta-\theta_{FA}(h)$$

$$\Delta q=q-q_{FA}(h)$$

$\theta_{FA}(h)$ and $q_{FA}(h)$, the potential temperature and humidity in the free atmosphere, can be obtained from atmospheric sounding (Suppl. Fig. S1).

**Figure A1**. Schematic of the ABL potential temperature and absolute humidity profiles.

Entrainment occurs as surface heating generates buoyant thermals that rise through the boundary layer. When these thermals reach the capping inversion, they overshoot and displace air from the free atmosphere, which is subsequently mixed into the ABL. The entrainment velocity is parametrized following Vilà-Guerau de Arellano et al., (2015) as:

$$w_{e}=\frac{1}{\Delta\theta_{v}}\left( \beta{\overline{w^{'}\theta_{v}^{'}}}_{s}+5u_{*}^{3}\frac{\theta_{v}}{gh} \right)$$

where ${\overline{w^{'}\theta_{v}^{'}}}_{s}={\overline{w^{'}\theta}}_{s}+0.51{\overline{w^{'}q^{'}}}_{s}$is the buoyant flux, $\theta_{v}$ is virtual temperature. The above expression includes the effects of mechanical turbulence at the surface via friction velocity $u_{*}$. Within this parametrization, the buoyant flux at the top is assumed to be a constant fraction $\beta$ of the flux at the surface.

In the surface layer, temperature and humidity profiles follow Monin-Obukhov similarity theory, which are computed from Eqs. (6), with aerodynamic resistances given by:

$$r_{aH}=r_{aq}=\frac{\log\left( \frac{z_{SL}-d_{0}}{H_{c}-d_{0}} \right)-\Psi_{H}\left( \frac{z_{SL}-d_{0}}{L} \right)+\Psi_{H}\left( \frac{H_{c}-d_{0}}{L} \right)}{0.4u_{*}}$$

where $z_{SL}$ is the height of the surface layer, assumed to be equal to 10% of ABL depth $h$ (Stull, 1988; Vilà-Guerau de Arellano et al., 2015), $\Psi_{H}$ is the stability correction function, $L$ is the Obukhov’s length and $d_{0}$ the displacement height (70% of canopy height). The stability correction function can be computed by (Garratt, 1994):

$\Psi_{H}\left( \zeta\right)=\left\{ \begin{aligned} 2\ln\left( \frac{1+\sqrt{1-16\zeta}}{2} \right) \zeta<0 \\ -5\zeta\zeta>0 \end{aligned} \right.$
